# Tunable Luminescence from a Single Free-Base Porphyrin Molecule by Controlled Access to Optically Active States

**DOI:** 10.1021/acsnano.6c01502

**Published:** 2026-07-20

**Authors:** Eve Ammerman, Nils Krane, Bruno Schuler

**Affiliations:** nanotech@surfaces Laboratory, 1666 Empa − Swiss Federal Laboratories for Materials Science and Technology, Dübendorf 8600, Switzerland

**Keywords:** Electroluminescence, Scanning Tunneling Microscopy, Charge Dynamics, Electronic Transitions, Tunnel Junctions, Molecules, Porphyrin

## Abstract

Scanning tunneling microscopy-induced luminescence (STML) provides access to the optical properties of individual molecules through a cascade of relaxation processes between many-body states. Insufficient charge attachment energies prevent relaxation via optically active states, causing even intrinsically bright molecules to remain dark in STML. Here, we utilize substrate work function control and tip-induced gating of the double-barrier tunnel junction to induce an energy shift of the ionic transition state of a single free-base tetrabenzoporphyrin (H_2_TBP) to control access to optically active states and bright exciton emission. The experimental observations are validated by a rate equation and a polaron model considering the relaxation energy of the NaCl decoupling layer upon charging of the molecule.

## Graphical abstract

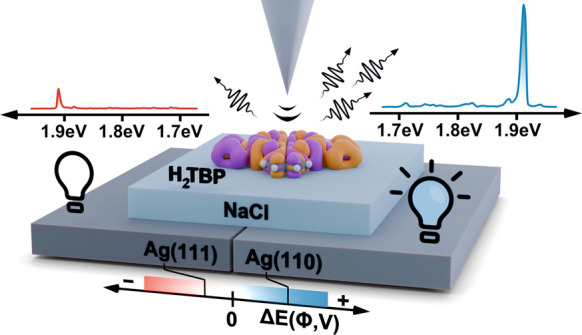

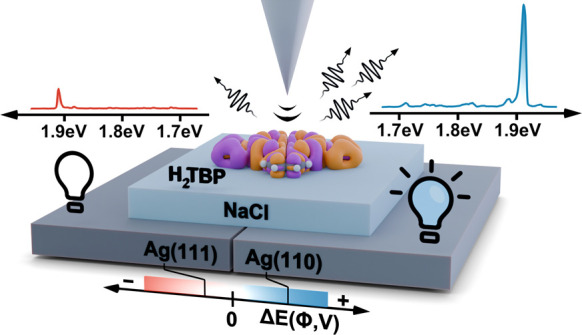

## Introduction

Understanding the optoelectronic (photophysical) properties of organic molecules, such as porphyrins, is technologically relevant for organic light emitting diodes (OLEDs) and organic photovoltaics,^1^ and fundamentally important for understanding biological systems, such as photosystem I & II.^2,3^ Recent developments in on-surface synthesis methods have led to the incorporation of porphyrins alongside π-radical magnetic nanographenes^4–7^ resulting in tailor-made molecular structures with engineered electronic, magnetic, and optical properties. Porphyrins offer unique opportunities for optical and spin tunability through metalation of the core and coordination chemistry, thereby enabling exciting prospects for designing the coupling in hybrid porphyrin-nanographene structures.

Scanning tunneling microscopy (STM) provides access to single molecules and orbital-resolved electronic measurements. Additionally, STM luminescence (STML) allows probing of their optical properties with characterization comparable to both fluorescence and Raman spectroscopy.^8–11^ Phthalocyanine (H_2_Pc) chromophores, structurally analogous to free-base tetrabenzoporphyrin (H_2_TBP), have become the benchmark system in STML studies.^9,10,12–17^ In contrast, nanoscale optical characterization of porphyrins remains surprisingly scarce^18–22^ despite their central role in surface science.^23^

Although H_2_TBP and H_2_Pc share a benzene-annulated tetrapyrrolic framework, differences in the macrocycle composition lead to distinct photophysics.^24^ In H_2_Pc, the meso positions are occupied by nitrogen atoms instead of carbon, increasing electronic delocalization and rigidity.^25^ Consequently, they exhibit different excited-state relaxation dynamics, although both families generally maintain high fluorescence quantum yields.^24^

Here we show that H_2_TBP is inherently dark in single-molecule electroluminescence on 3 ML NaCl/Ag(111) due to unfavorable energy alignment. H_2_TBP has a smaller hole attachment energy than the chemical analog H_2_Pc, suppressing STML emission by ∼98% compared to H_2_Pc on Ag(111). By locally tuning the electrostatic environment, either via substrate work function or tip-induced gating in a double-barrier tunnel junction, we can overcome this limitation; accessing otherwise forbidden optical transitions and activating bright emission from individual H_2_TBP molecules.

## Results and Discussion

### Molecular Ionic Transition State Energy on Few-Layer NaCl

STML measurements are performed on individual H_2_TBP and H_2_Pc molecules decoupled from a Ag(111) substrate by 3 ML insulating NaCl islands, as sketched in Figure 1a. We will refer to these two systems as TBP_111_ and Pc_111_ in the following. Figure 1b shows the corresponding STML spectrum of Pc_111_ (black) recorded with an excitation rate of 63 pA at −2.5 V and an acquisition time of 30 s. The dominant peak at 1.81 eV and its lower energy vibronic satellites can be assigned to the S_1_ (or *Q*_*x*_) emission line and the weaker spectral lines around 1.94 eV to the S_2_ (or *Q*_*y*_) emission, in agreement with the literature.^13^ The STML spectrum on the nearby H_2_TBP molecule (orange), taken with the same tip and on the same NaCl island, shows a peak at 1.91 eV, which we assign to S_1_, and no emission at higher energy could be detected. Strikingly, the emission intensity of TBP_111_ is significantly weaker than that of Pc_111_ emission, necessitating a higher excitation current (150 pA) and relatively long integration time (120 s). After normalizing the luminescence spectra to photon counts per tunneled electron, the TBP_111_ spectrum has an intensity 50 times lower than that of the Pc_111_ spectrum. Such a low photon yield of TBP_111_ poses a significant challenge for experiments requiring high sensitivity, such as high-resolution fluorescence mapping,^10^ detection of weak vibronic satellite emission,^14,26,27^ or potential phosphorescence on metalated porphyrins.^28^

**Figure 1:**
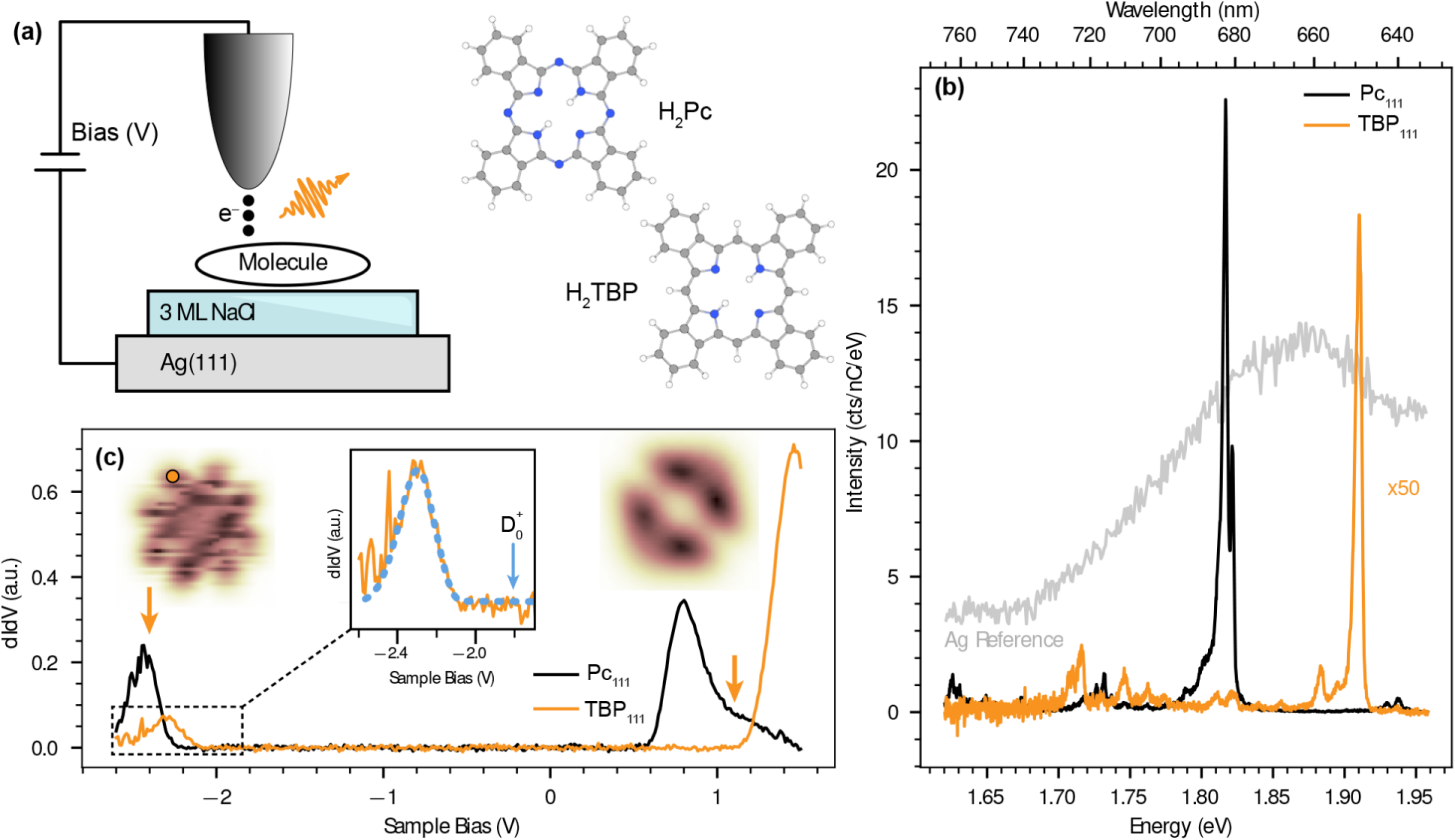
Electronic and optical fingerprints of H_2_TBP (a) Cartoon of STM luminescence experiments of Pc_111_ and TBP_111_ adsorbed on a thin-film NaCl decoupling layer and supported by Ag(111). (b) Comparison of fluorescence intensity from single TBP_111_ and Pc_111_ molecules. STML of TBP_111_ measured at −2.3 V and Pc_111_ measured at −2.5 V. (c) Scanning tunneling spectroscopy of H_2_TBP shown with comparison to Pc_111_ to emphasize the upward shift of the molecular transport resonances. Constant-height STM images show the spatial distribution of the PIR and NIR recorded at −2.4 and 1.1 V, respectively. In both STS measurements, feedback was opened at positive sample bias with a set point current of 40 pA. The STS and STML data in panels b and c were recorded from molecules adsorbed on the same NaCl island with the same tip to ensure fair comparison of optical intensity. The inset in panel c shows a polaron model fit to the PIR, yielding a $D_{0}^{+}$ state energy of 1.81 eV.

To explain the STML excitation mechanism and dark emission of TBP_111_, we adopt the many-body description of molecular electronic states which was first introduced by Miwa et al. and is now a well-established practice that provides a comprehensive description of electronic excitations and optical emission arising from different charge states.^29–32^ In the many-body framework, the energy level alignment between a transient charge state and the optically excited state should be considered to be the primary factor restricting fluorescence of TBP_111_. Scanning tunneling spectroscopy (STS) measurements of TBP_111_ and Pc_111_ (Figure 1c) were recorded at the same tip positions as the STML spectra in Figure 1b. The STS spectra reveal a pronounced upward shift of the positive and negative ion resonances of TBP_111_ relative to those of Pc_111_, accompanied by an increased transport gap. This shift can be attributed to the presence of four additional electronegative nitrogen atoms in the H_2_Pc macrocycle.

At first glance, the observed shift does not appear to limit electro-excitation of TBP_111_. The onset of the positive ion resonance (PIR) in the STS spectra appears at an applied bias of −2.1 V suggesting a nominal energy of 2.1 eV of the positively charged molecule $\left(D_{0}^{+}\right)$ upon hole injection. This energy lies approximately 200 meV above the optical emission line at ∼1.91 eV, seemingly satisfying the energy requirement for electroluminescence. However, this interpretation neglects the relaxation processes accompanying molecular charging on the surface. In particular, the ionic NaCl surface has been shown to substantially contribute to the reorganization energy of the system during charge injection into the molecule.^33,34^ This high electron–phonon coupling of the NaCl environment leads to vibronic excitation and, consequentially, to an apparent broadening and shift in energy of the ion resonances in STS. The energetic shift is comparable to the charge-state relaxation energy, λ_+_, as illustrated in Figure S1. To obtain the true zero-phonon energy of $D_{0}^{+}$ , the precise onset of the ion resonance peak needs to be determined, which is experimentally challenging. At such high electron–phonon coupling, the zero-phonon transition becomes vanishingly small, due to an effective Franck–Condon blockade^35^ and increasing the tunneling current signal by approaching the tip risks a picking-up of the molecule. Vasilev at al. demostrated an alternative method to determine the true ground-state energy of a charged molecule on NaCl by utilizing polaron theory to model the line shape of the broadened resonance.^27^ We fitted the experimental STS spectra with the polaron model adapted from ref 27. and obtained a $D_{0}^{+}$ energy of 1.81 eV for TBP_111_, after accounting for a voltage drop of 10%. For Pc_111_, the $D_{0}^{+}$ energy was found to be of 1.98 eV (more details in the SI). The results suggest the $D_{0}^{+}$ energy of Pc_111_ is about 200 meV higher than its S_1_ state at 1.81 eV, enabling the $D_{0}^{+}\to S_{1}$ transition and, accordingly, bright emission from Pc_111_. On the other hand, for H_2_TBP, the $D_{0}^{+}$ energy is ∼100 meV lower than the respective S_1_ state and emission should be fully quenched. To explain the experimentally observed STML intensity, one needs to consider the dielectric and geometric properties of the STML tunnel junction.

### Tuning the Local Electrochemical Potential

In a double-barrier tunneling junction (DBTJ) geometry, a fraction of the applied bias voltage drops across the insulating film, leading to a shift in the electrochemical potential (Δ*E*) of the adsorbed molecule with respect to the substrate.^36^ For molecules on few-layer (3–4 ML) NaCl, a voltage drop of α ∼10% can be estimated.^27,29,37^ The STML measurements are typically performed with applied absolute voltages in the range of 2–3 V providing an effective gating voltage of 200–300 meV, which is on the order of the energy difference between the TBP_111_ $D_{0}^{+}$ and S_1_ states. Hence, this gating effect is expected to lift the $D_{0}^{+}$ state energy across the transition threshold to S_1_ leading to an emission intensity strongly dependent on the applied bias.

Experimentally, the effect can be observed by taking STML spectra at different bias voltages but constant tip height, to keep the tip’s nanocavity plasmon unchanged.^30,32^ With increasing absolute bias voltage, more vibronic tunneling channels from S_0_ to $D_{0}^{+}$ are continuously opened, leading to an increase in current and, accordingly, STML intensity.^29^ To account for this effect, the STML intensity was normalized by the total current. The normalized photon counts per tunneled electron emission intensities of the S_1_ line were integrated between 642 nm and 652 nm and are displayed in Figure 2c. Instead of a sharp step like increase, once the gating voltage is lifting $D_{0}^{+}$ energetically above S_1_, there is a steady increase in intensity with applied bias voltage. This dependence can be understood when we examine the relative transition rates between the $D_{0}^{+}$ charge state and the optical state S_1_. Figure 2a illustrates the many-body state diagram relative to the ground state (S_0_). As described above, the $D_{0}^{+}$ state energy on Ag(111) lies about 100 meV below the S_1_ state without any bias applied, preventing the transition and subsequent radiative relaxation. With a negative bias voltage applied to the STM junction $D_{0}^{+}$ is shifted upward by Δ*E*(*V*) due to the voltage drop, enabling access to the $D_{0}^{+}\to S_{1}$ transition. Analogous to the $S_{0}\to D_{0}^{+}$ transition, $D_{0}^{+}\to S_{1}$ involves a charge change of the molecule that is subject to the strong electron–phonon coupling of the NaCl layer. The transition step is accordingly shifted to higher energies and broadened, leading to the steady increase in intensity with bias voltage, as the continuous shift of $D_{0}^{+}$ steadily increases the transition rate into S_1_.

**Figure 2:**
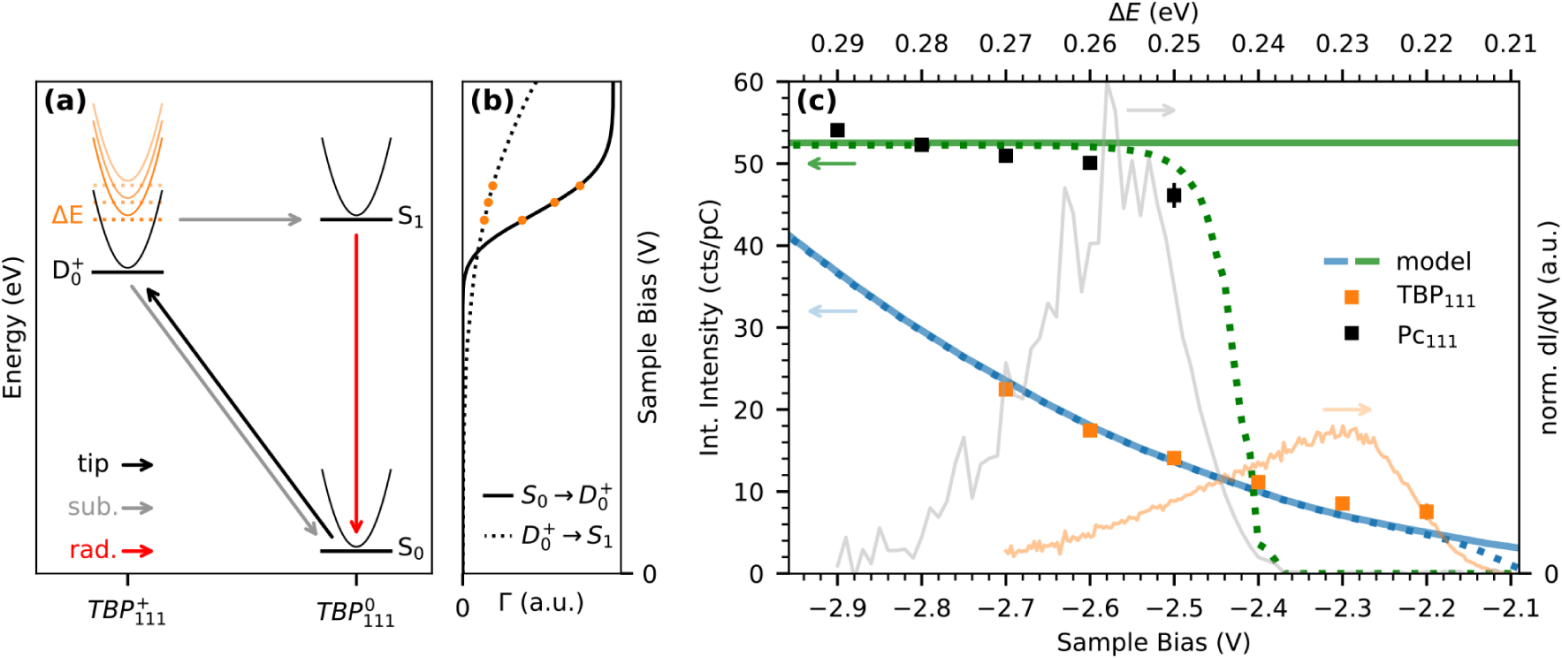
Local tip gating in single molecule fluorescence (a) Many-body diagram describing the electronic states and transitions of a TBP_111_ molecule during STML experiments. Black and gray arrows connecting different TBP_111_ charge states represent electron transfer involving the tip and substrate while the red arrow represents radiative relaxation. The excess relaxation energy needed for charge transport is depicted as a parabola above each state. A voltage dependent shift in $D_{0}^{+}$ energy, Δ*E*(*V*), is present during experiments and indicated by a series of orange dashed lines. (b) Voltage dependent $S_{0}\to D_{0}^{+}$ and $D_{0}^{+}\to S_{1}$ transition rates. Orange circles correlate with Δ*E*(*V*) shown in (a). (c) [left axis] Integrated intensity of the S_1_ spectral feature for both TBP_111_ and Pc_111_. Spectral intensity is normalized by the tunneling current and integration time. The raw spectra and integration ranges are shown in supplementary Figure S2. A model simulation of the voltage dependence is shown for TBP_111_ in blue and Pc_111_ in green. A dashed colored line represents the model simulation with a dark background current of 150 fA that does not contribute to the emission but reduces the observed emission rate at low currents. [right axis] Normalized dI/dV of TBP_111_ and Pc_111_ PIR shown for reference. For the d*I*/d*V* measurements, the STM feedback was open with a set point sample bias of −2.7 V and current −50 pA for TBP_111_ and −2.9 V and −30 pA for Pc_111_.

Using a simple rate equation model,^32^ we simulated the voltage dependent emission rate to reproduce the experimental observation and strengthen the gating model. For this, the voltage-dependent transition rates $\Gamma_{S_{0}\to D_{0}^{+}}$ and $\Gamma_{D_{0}^{+}\to S_{1}}$ were calculated with the polaron model described above. The critical role of the polaron model is to capture the vibronic response of the NaCl upon (dis)­charging of the molecule and provide a voltage dependent spectral function *S*(*V*)∝ dΓ/d*V* that can be used to describe the transition rates Γ­(*V*). The STS measurement of the positive ion resonance largely reflects the voltage dependence of the transition rate derivative between S_0_ and $D_{0}^{+}$ , thus, $\Gamma_{S_{0}\to D_{0}^{+}}$ can be well approximated by the integral of the polaron spectral function fitted to the experimental d*I*/d*V*. However, there is no direct measure of the gating dependent transition between the charge state and S_1_. As an approximation, we assumed the relaxation energy λ_+_ for both transitions to be the same and described them with the same spectral function, depending on the effective applied voltage between molecule and corresponding lead, i.e., *S*((1-α)*V*_*b*_) and *S*(-α*V*_*b*_), respectively. The resulting voltage dependent transition rates are sketched in Figure 2b and Figure S7. Because both the tip height and lateral position strongly influence the local voltage drop and tunneling rate, these two quantities were treated as fitting parameters in the model. The resulting shift in the electrochemical potential Δ*E* (top axis of Figure 2c) scales with a voltage drop of 10.2% which is within the expected range of 10–15%. More details on the model and its implementation are provided in the SI including a detailed analysis regarding the voltage drop dependence of the model. The resulting simulated voltage dependence of the emission rate (blue line in Figure 2c) is in good agreement with the experiment (orange dots).

To validate the gating model, we also recorded the voltage dependent emission of Pc_111_ (black dots in Figure 2c). Contrary to TBP_111_, the normalized intensity remains near constant with the bias voltage. Due to the higher ion resonance energy of Pc_111_, the $D_{0}^{+}\to S_{1}$ transition is almost ″fully opened″ and the STML intensity is only limited by the initial $S_{0}\to D_{0}^{+}$ transition. In the typical STM regime, the latter is proportional to the tunneling current and normalizing the STML intensity with current will yield a near constant signal, as observed in experiment. Please note, for very small currents, i.e., at bias voltages >−2.6 V where tunneling into the ion resonance is limited, one needs to consider the effect of a small background current *I*_bg_, flowing directly from the sample toward the tip. It skews the normalization of the STML intensity, as the additional background current becomes significant for very small total currents in the denominator of the normalization *J*_ph_/(*I*_mol_ + *I*_bg_) and causes an apparent downshift of the STML intensity. To capture this effect in our model, we added a constant offset of 150 fA to the simulated current *I*_mol_ (dotted lines in Figure 2c).

In the following, we discuss the applicability of other excitation mechanisms, reported in the literature. Direct plasmonic excitation has been reported for molecules on thin decoupling layers, where inelastic tunneling directly between tip and substrate can transfer energy to the molecule, mediated by the plasmonic states in the nanocavity.^14^ The process requires sufficiently high currents between the tip and substrate and has, for this reason, been shown for bias voltages within the electronic gap of the molecule. For TBP_111_, the effect can be ruled out for two reasons: on the one hand, there is little to no current flowing directly between tip and substrate, as can be seen by the vanishing current before the onset of the $D_{0}^{+}$ . On the other hand, plasmonic excitation should occur already at absolute bias voltages larger than the photon energy of 1.91 V, which has not been observed in the experiment. Another excitation mechanism could be electronic pumping via the triplet state *T*_1_ into either a higher positive ion resonance or a negatively charged state.^32,38^ These processes require tunneling of two electrons between the tip and molecule to reach S_1_ and are expected to be much smaller than the single-electron gating model explained above. Electronic pumping alone could not be fitted with the rate equation model to the experimental data within a physically reasonable parameter space.

To recapitulate, during our STML measurements two electronic excitations are required in order to access the optically active states, $S_{0}\to D_{0}^{+}$ and $D_{0}^{+}\to S_{1}$ . Each excitation can be treated as independent charge state transition and both are subject to a resonance shift and broadening due to the underlying NaCl layer. Therefore, despite a shift of Δ*E* > 200 meV for bias voltages at the onset of the PIR, luminescence remains below the experimental detection limit. It is not sufficient to simply raise the $D_{0}^{+}$ energy above S_1_ but a larger shift is needed to compensate for said resonance shift and broadening. Extrapolation of the rate equation model to more negative bias voltages shows, that the emission intensity of TBP_111_ on Ag(111) is still quenched by 94% for bias voltages around −2.5 V. The model indicates a bias voltage <−5.6 V, Δ*E* ≥ 560 meV, is needed to achieve optimal energy level alignment. Applying such a large bias voltage in an experiment would lead to unwanted effects, including a transition from the tunneling regime to field emission which would result in a loss of spatial resolution and risk damage to the molecule being studied.

### Engineered Energy Level Alignment for Optical Characterization

For reasons previously mentioned, the chemical potential shift induced by the voltage drop in the STM junction provides only limited tuning range. The local gating effect is insufficient to fully overcome the energetic constraint limiting the $D_{0}^{+}\to S_{1}$ transition, as shown by the strong voltage dependence of the emission intensity. The effective voltage drop could be increased by introducing a thicker decoupling layer. However, the increased lever arm of the gating potential would be even more sensitive to the tip position relative to the molecule, making the understanding of experiments that include spatially mapping the STML intensity more complex. This effect can already been seen for 3 ML as discussed below. To extend the tuning range of the chemical potential, we exploit the intrinsic energy level alignment defined by the substrate work function (Φ), which enables a static shift of the molecular ion resonances. To this end, we performed STML measurements of H_2_TBP on NaCl films deposited on Ag(110) with a thickness of ∼598 pm, which will be referred to as TBP_110_. The decoupling layer thickness was chosen to achieve similar charge state lifetimes, and ensure comparable decoupling, between TBP_110_ and TBP_111_ (see Figure S10). The Ag(110) substrate retains the strong plasmonic enhancement of silver while reducing the work function from Φ_Ag111_ = 4.5 eV to Φ_Ag110_ = 4.1 eV.^39^ Lowering the workfunction by 400 mV is expected to shift the positive ion resonance energies of adsorbed molecules by a similar amount to higher energies. This shift is confirmed experimentally by comparing the STS spectra of decoupled H_2_TBP on Ag(111) and Ag(110), with the PIR on Ag(110) being shifted by 400 meV to −2.5 eV (Figure 3a). A polaron model fit of the STS spectra of TBP_110_ (Figure S1b) yields a $D_{0}^{+}$ energy of 2.25 eV, matching the work function shift. The shift in the $D_{0}^{+}$ state energy provides approximately ∼300 meV excess energy relative to the S_1_ state, comparable to the $D_{0}^{+}\to S_{1}$ transition of Pc_111_. Correspondingly, STML measurements of TBP_110_ reveal a significant increase in the emission intensity and the appearance of an additional emission line at 2.15 eV, or 230 meV above S_1_ (Figure 3b). The new emission line can be tentatively assigned to S_2_, which has been reported to be ∼150 meV above S_1_ for absorption spectroscopy in vitrified solution.^40^ Another origin of the emission line could be hot-luminescence, where a vibrationally excited molecule emits while relaxing into the vibrational ground state.^22,41^

**Figure 3:**
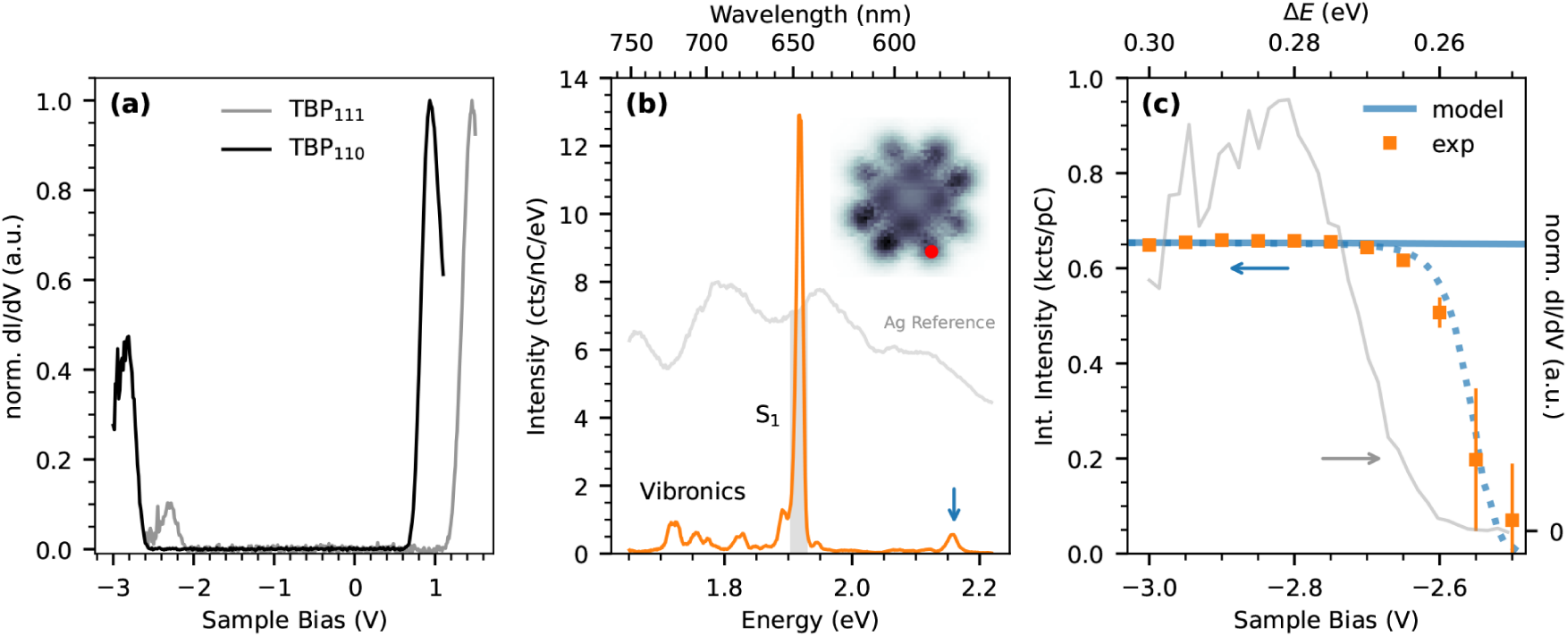
Engineered energy level alignment for enhanced optical characterization. (a) Comparison of STS measurements for TBP on NaCl with Ag(111) and Ag(110) as the conducting substrates. The STS measurement with Ag(110) was recorded with the feedback opened with a sample bias of −3 V and current of −55 pA. (b) STML spectra of a TBP_110_ molecule. Measurement performed with *V* = −3.0 V, *I* = −314 pA and *t*_acq_ = 15 s. A reference spectrum recorded on the Ag surface at *V* = −3.0 V is shown in gray. An inset with a constant-height STM image indicates where the spectrum was recorded. A blue arrow indicates the observation of a new spectral feature at 2.16 eV. (c) [left axis] Integrated intensity of the S_1_ spectral feature indicated in (b). Spectral intensity is normalized by the tunneling current and integration time. The raw spectra and integration ranges are shown in supplementary Figure S2. A model simulation of the voltage dependence is shown for TBP_110_ in blue. A dashed colored line represents the model simulation with a dark background current of 150 fA. [right axis] Normalized dI/dV of the TBP_110_ PIR shown for reference. Error bars are provided to show the rapid decrease in signal-to-noise when transport through the PIR is minimal.

A direct comparison of emission intensity with H_2_Pc is not possible on the NaCl/Ag(110) substrate, since the reduced work function causes a charging of H_2_Pc and thus prohibits a reliable comparison. To corroborate the higher STML efficiency of TBP_110_ compared to TBP_111_ we again performed voltage dependent STML spectroscopy. In Figure 3c, the voltage dependence of the normalized emission intensity is shown. It exhibits a clear plateau as soon as the applied voltage reaches the onset of the PIR, in contrast with the observations for TBP_111_, where the emission increases gradually, even at voltages significantly beyond the PIR (Figure 2c). By engineering the level alignment we shift the $D_{0}^{+}$ sufficiently high in energy that the $D_{0}^{+}\to S_{1}$ transition becomes ″saturated″ and does not increase anymore with more negative bias voltages (Figure S4). Applying the rate-equation model to the STML data reproduces an approximately constant emission rate for S_1_, similar to the one observed for Pc_111_.

### Gating Effects in Spatially Resolved Fluorescence Maps

A significant benefit of performing STML measurements on single molecules is the angstrom-scale spatial resolution provided by the technique. In hyper-resolved fluorescence mapping (HRFM), many fluorescence spectra are recorded on a dense grid across an individual molecule. Analysis of the intramolecular spatial distribution of spectral features can help identify the origin of emission lines and provide insight into the photophysics and vibronic fine structure of molecules with distinct structure and adsorption geometries.^14,42,43^

Time-dependent density functional theory (TD-DFT) calculations of the H_2_TBP excited states predict an S_1_ transition dipole along the axis *A*_*x*_, defined by the two inner hydrogens of the macrocycle (see Figure S13), while the energetically higher state, S_2_, has a transition dipole along the orthogonal *A*_*y*_ axis, analogous to H_2_Pc. In the experiment, we expect a mixing of the axes due to fast tautomerization of the two central hydrogen atoms. Previous STML studies of H_2_Pc report that an NaCl substrate-induced anisotropy can lift the degeneracy between the corresponding excited states S_1,*x*_ and S_1,*y*_.^10,43^ This splitting is reflected in the double-peak structure of the main emission line of Pc_111_ shown in Figure 1b.

A similar splitting of the two states can also be observed for TBP_110_. Figure 4a shows two emission spectra taken along the *A*_*x*_ and *A*_*y*_ axes of TBP_110_ exhibiting a relative lineshift of ∼10 meV. Utilizing the splitting of the emission lines, we can map the spatial intensity distribution of S_1,*x*_ and S_1,*y*_, by integrating the spectral intensities on the high and low energy flanks of the primary emission lines (see gray areas in Figure 4a). The resulting fluorescence maps, normalized by excitation current, are shown in Figure 4b,c. They both feature a nodal plane with orthogonal symmetry axes and maximal emission intensity near the periphery of the molecule. These experimental HRFM maps are well reproduced by TD-DFT calculations of the corresponding excited states, including the plasmonic response of the STM tip in the simulation (see Figure 4f,g and Figure S14a).

**Figure 4:**
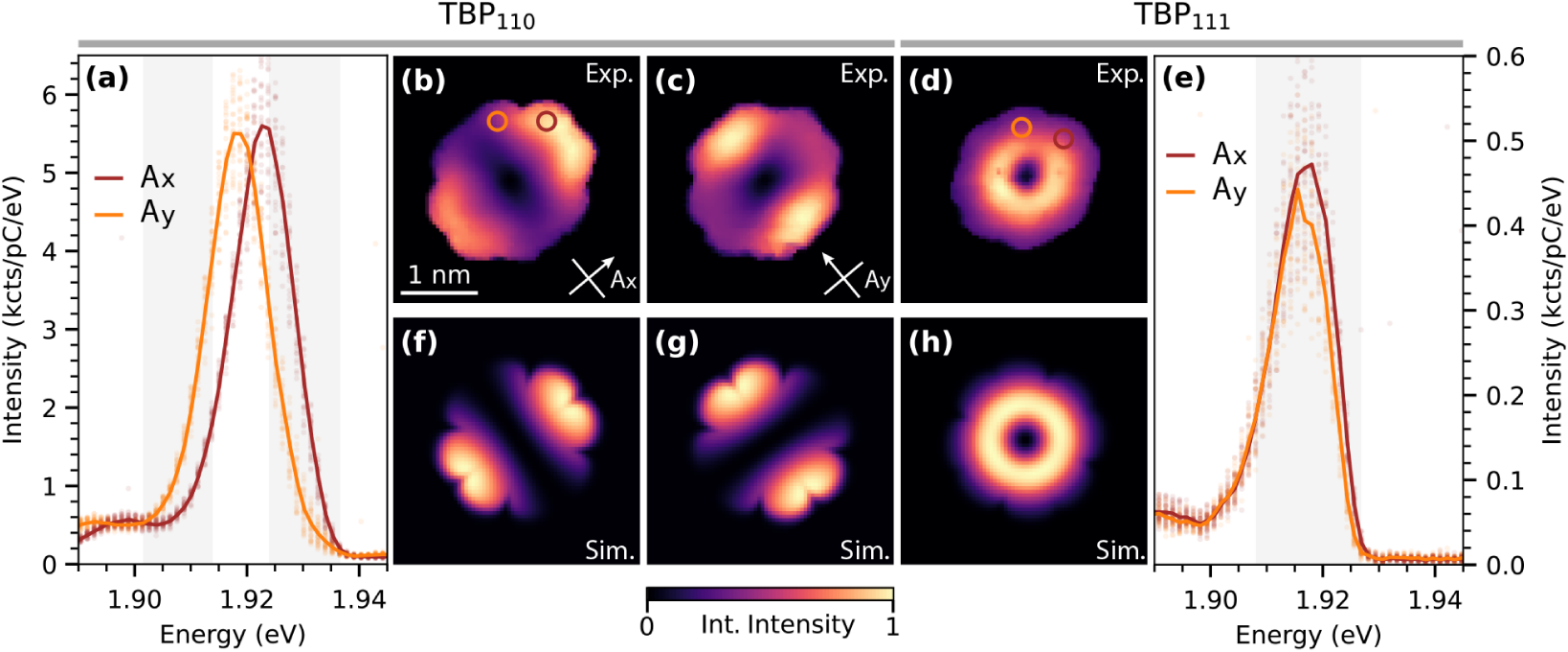
Fluorescence mapping of the S_1_ emission line of H_2_TBP (a) STML spectra from the *A_x_* and *A_y_* axes of TBP_110_, at locations indicated in (b). (b, c) S_1_ constant height fluorescence maps of the two tautomer states of TBP_110_ between 1.923 and 1.937 eV and 1.902–1.914 eV, respectively. The orientation of the optical state matches the *A_x_* and *A_y_* symmetry axes of the molecule as indicated by the white arrows. (d) Constant height fluorescence map of TBP_111_ between 1.907 and 1.927 eV. (e) TBP_111_ reference STML spectrum from the *A_x_* and *A_y_* axes. (f, g) Simulated STML maps of S_1_ of the TBP_110_ tautomers. (h) Simulated STML map of the TBP_111_ molecule. STML map simulations were created using the polaron and rate model described in the main text with a spatially dependent voltage drop described in the supplement. All STML map intensities are normalized to 1 and share the same scale bar. The TBP_110_ map used for (b) and (c) was recorded at −2.8 V and *t*_acq_ = 15 s. The TBP_111_ map used for (d) was recorded at −2.4 V and *t*_acq_ = 30 s. The *A_x_* and *A_y_* reference spectrum are averages of 29 measurements that were recorded within a 6 pixel diameter indicated by the colored circles in (b) and (d). The integration regions used to generate fluorescence maps in are indicated with gray boxes in (a) and (e). Postprocessing of the experiential STML maps, (b–d), with a 3 × 3 median filter is performed to remove the effect of cosmic rays and improve image quality.

For TBP_111_, in contrast, we do not observe a splitting of the two tautomeric excited states. The STML spectra shown in Figure 4e exhibit a slight intensity difference between the *A*_*x*_ and *A*_*y*_ directions, likely reflecting small variations in the tautomer lifetime, but no distinct lineshift. The HRFM of the integrated primary emission line (gray area in Figure 4e) is shown in Figure 4d. Consistent with the near degeneracy of S_1,*x*_ and S_1,*y*_, the map does not exhibit the 2-fold symmetry that was observed for TBP_110_, but instead displays a ring-like intensity distribution. The stronger splitting of S_1,*x*_ and S_1,*y*_ for TBP_110_, compared to TBP_111_, can be rationalized by the stronger anisotropy of the NaCl/Ag(110) interface, where significant structural anisotropy of the NaCl layer has been reported.^44^

Besides the tautomerization effects discussed above, comparison between the HRFM of TBP_110_ and TBP_111_ shows an additional difference in the spatial distribution of emission intensity. For TBP_110_, the regions of highest normalized intensity are located at the edges of the map, whereas for TBP_111_ the S_1_ emission forms a ring close to the center of the molecule. Since fluorescence maps primarily reflect the coupling between the plasmonic nanocavity and the excited-state transition density^10,45^ both systems would *a priori* be expected to exhibit similar spatial profiles. To understand the difference, we need to consider the effect of the voltage drop, and subsequent gating, that significantly influences excitation efficiency in TBP_111_. The nonlinear behavior shown in Figure 2c is intrinsically related to the energy shift of the $D_{0}^{+}$ state, which is proportional to the voltage drop, and is highly sensitive to the tip position.^46,47^ The tip-induced gating of the molecule is highest when the tip is placed above the center of the molecule and decreases when the tip is moved radially away from the molecule center. In the case of TBP_111_, the reduced gating potential when the tip is placed at the edge of the molecule limits the $D_{0}^{+}\to S_{1}$ transition rate and significantly reduces the emission intensity. On the other hand, the transition density of H_2_TBP does not couple to the tip plasmon when the tip is centered above the molecule. These two effects taken together lead to vanishing intensity in both the center of the molecule as well as at the edges, leading to a ring shaped emission profile observed in the TBP_111_ fluorescence maps.

To corroborate our explanation of the observed differences in the spatial profile of STML emission of TBP_111_ and TBP_110_, we adapt our polaron and rate equation model to spatially resolved STML map simulations by expanding the voltage drop to include a tip-position dependency. We assume a simple two-point-charge model to describe the spatial dependence of the gating^48^ and the coupling to the plasmon^45^ (see SI for details). The simulated fluorescence map for TBP_111_, including fast tautomerization effects, is shown in Figure 4h, reproducing the experimentally observed ring-shaped intensity profile.

## Conclusions

In summary, we have shown that tuning the hole attachment energy via tip-induced gating or by controlling the substrate work function enables efficient electrical excitation into optically active states from single H_2_TBP molecules. Quantitative analysis of the hole attachment energy using a polaron model highlights the critical role of the relaxation energy of the NaCl decoupling layer in determining the energy requirements for transitions to the excited states. We demonstrated the energetically restricted access to the optically excited state S_1_ for TBP_111_ by voltage-dependent STML emission, which could be modeled by rate equations that consider the NaCl relaxation energy extracted from STS. Comparison of TBP_111_ and TBP_110_ fluorescence maps clearly demonstrates the significance of the local gating effect and the impact it has on the spatial characterization of electroluminescence from single molecules. Controlled energy-level engineering in STML enables a detailed, quantitative probe of molecular photophysics in regimes where the ionic transport energy and optical excitations are comparable, offering a general strategy for studying chromophores at the single-molecule level.

## Methods

### Sample Preparation

Sample preparation of H_2_TBP and H_2_Pc decoupled from Ag(111) and Ag(110) by thin-films of NaCl. Sputter and anneal of single-crystal silver substrates by repeated argon ion bombardment and heating to 673 K. Growth of NaCl thin-films on Ag surfaces at room temperature by sublimation from a Knudson-type effusion cell at 740 °C and postannealing to 200 °C for NaCl on Ag(111) and 230 °C for NaCl on Ag(110). The difference in NaCl thickness is chosen to achieve comparable topographic heights in STM measurements of ∼600 pm and comparable charge state lifetimes (see Figure S10). In-situ flash-deposition of molecules was performed from a direct-current Si heater with direct line of sight to the STM scan head held at 4.5 K.

### STML Measurements of Single Molecules

SPM measurements were performed using a CreaTec Fischer & Co. GmbH scanning probe microscope at liquid helium temperatures (*T* < 5 K) under ultrahigh vacuum (*p* < 2 × 10^–10^ mbar). STM and STS measurements performed with a FEMTO-DLPCA200 preamplifier with a gain of 10^9^ A/V. Light collection from the STM tunnel junction is done with a silver off-axis parabolic mirror that has a focal length of 33.8 mm and a diameter of 1 in. with an incident angle of 60 degrees and an estimated numerical aperture of 0.35. Optical spectra were collected through a fiber collimator using a Teledyne Princeton Instruments SpectraPro HRS 300 spectrograph, equipped with an LN-cooled PyLoN 400BR eXcelon detector with an estimated spectral resolution of ∼4 nm and ∼2 nm after diffracted from a grating of 150 l/mm (Figure 2c, Figure 3b/c, Figure 4) and 600 l/mm (Figure 1b), respectively. An Ag wire tip was used for all STM and STML measurements and was prepared by repeated indentations into the metallic substrate and voltage pulses. The optical response of the STM tip was optimized by repeated indentation until a plasmonic emission spectrum measured on the bare Ag surface at +2.5 V on Ag(111) or −2.5 V on Ag(110) overlapped with the spectral emission of the H_2_TBP molecule. Each STML emission spectrum is normalized by the tip-specific plasmonic response present at the time of measurement to account for intensity variations of the junction response. The hyper-resolved fluorescence mapping in Figure 4b–d was performed at constant height with a drift correction procedure. Drift correction was performed in constant-current conditions on the NaCl surface with a set point current of 10 pA and sample bias of 1.4 V (TBP_111_) and 1.0 V (TBP_110_). The fluorescence maps were recorded with a constant tip-height offset relative to the NaCl surface of ΔZ = 450 pm (TBP_111_) and 437 pm (TBP_110_). The fluorescence maps have a pixel-to-pixel distance of 50 pm and are restricted to locations near the molecule where excitation currents were greater than 0.2% of the maximum current (∼300–500 fA).

### Time-Dependent Density Functional Theory

Density functional theory (DFT) and time-dependent DFT (TD-DFT) calculations were performed in gas-phase using B3LYP functional and 6–31G* basis set within the Gaussian16 software package.^49^ TD-DFT used the Tamm–Dancoff approximation (TDA) and the emission spectra were simulated for a Lorentzian line shape with broadening of 40 cm^–1^ half-width at half-maximum. All calculations were performed in gas phase.

## Supplementary Material



## Data Availability

The data that support the findings of this study are available from the corresponding author upon request.

## Notes

Voltage dependent STML spectra; detailed description of polaron and rate equation model; decoupling properties of NaCl on Ag(111) and Ag(110) (PDF)
