# Supplementary material for: Tunable Luminescence from a Single Free-Base Porphyrin Molecule by Controlled Access to Optically Active States
Source: ACS Nano. 2026 Jul 20;20(30):21054–62. doi: 10.1021/acsnano.6c01502 (PMC13450429; doi:10.1021/acsnano.6c01502)
Supplement: Supplementary file 1 [file nn6c01502_si_001.pdf]

Supporting Information: Tunable Luminescence From a Single Free-Base Porphyrin Molecule By Controlled Access to Optically Active States

Eve Ammerman[1,*] Nils Krane[1] and Bruno Schuler[1]

[1] *nanotech@surfaces Laboratory, Empa – Swiss Federal Laboratories for Materials Science and Technology, Dübendorf 8600, Switzerland*

(Dated: July 10, 2026)

CONTENTS

Polaron Model 2

Voltage Dependent STML Intensites 4

Rate Equation Model for Voltage Dependent Emission 5

Role of Voltage Drop in Simulation 10

NaCl Decoupling on Ag(111) and Ag(110) 15

Simulation of Fluorescence Maps 16

References 21

POLARON MODEL

The observed dI/dV peaks of molecules on NaCl are known to be, both, broadened and shifted to higher absolute energy, due to the strong electron-phonon coupling of the underlying NaCl [1, 2]. To extract the zero-phonon ion resonance energy, we employ a simple polaron model, as described by Vasilev et al. [3]:

$$S(E_{fi}, Q_{fi}, V) = \frac{1}{2\pi} Re \left\{ \int e^{-ieVt/\hbar} \, e^{\mathcal{F}(E_{fi}, Q_{fi})} \, \mathrm{d}t \right\}, \quad \text{with} \tag{S1}$$

$$\mathcal{F}(E_{fi}, Q_{fi}) = -iQ_{fi}E_{fi}t/\hbar + \int_0^\infty J(\Omega) \, e^{-iQ_{fi}\Omega t/\hbar} \, \mathrm{d}\Omega, \tag{S2}$$

where E_{fi} is the energy difference between final and initial state and Q_{fi} a unitless charge number, being $+1$ or -1 for hole or electron attachment, respectively. The electron-phonon coupling strength of the NaCl is included in the rectangular function $J(\Omega)$, which we assume to have a constant value η between $\Omega_{\mathrm{min}} = 18\,\mathrm{meV}$ and $\Omega_{\mathrm{max}} = 31\,\mathrm{meV}$ and is zero otherwise [3].

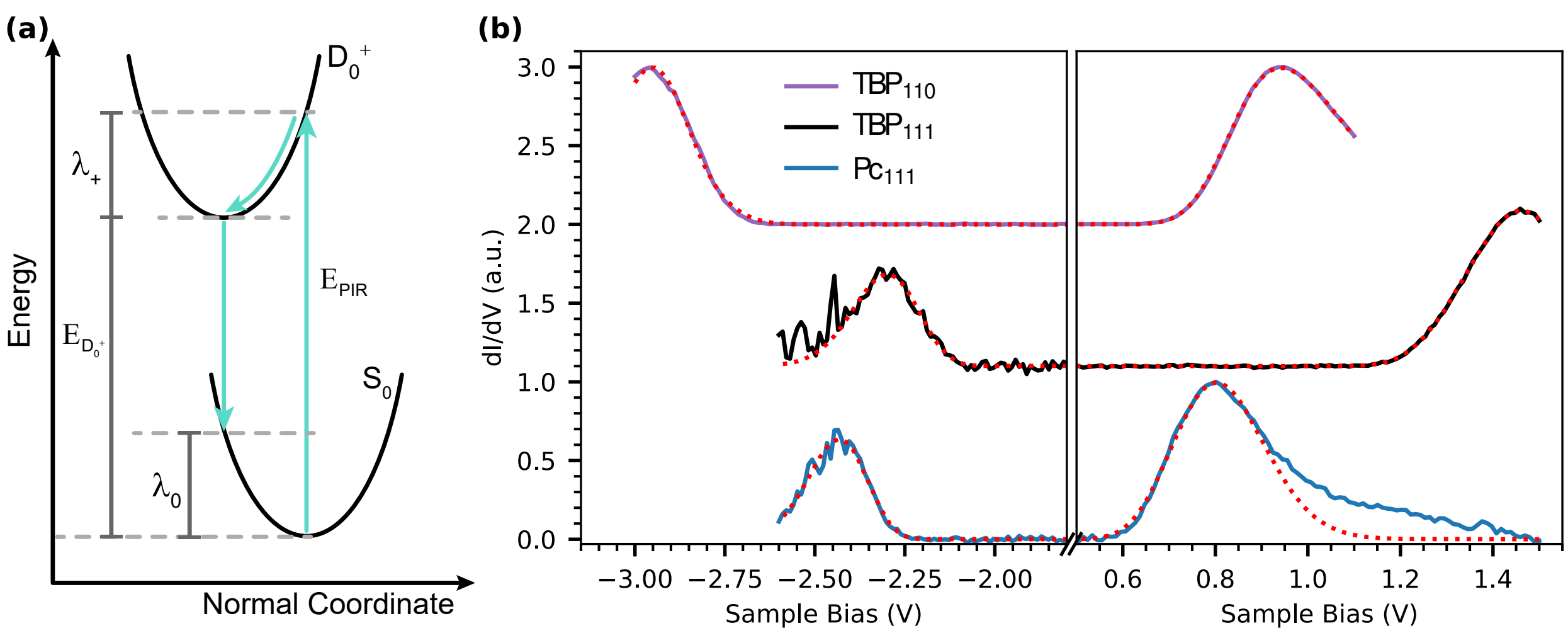

FIG. S1. **Polaron Modeling of dI/dV measurements.** (a) Sketch of the free-energy curves of a neutral and positively charged molecule on NaCl. The relaxation energy for ionization and neutralization are labeled λ_+ and λ_0, respectively. (b) Experimental dI/dV spectra taken on TBP on NaCl(4ML)/Ag(110) (purple) and NaCl(3ML)/Ag(111) (brown) as well as Pc on NaCl(3ML)/Ag(111). Dotted red lines correspond to the model fit with respect to the corresponding dI/dV data.

The parameters E_{fi} and $\lambda_\pm$ are used as fit parameters for the polaron model to the dI/dV

spectra. Here, E_{fi} corresponds to the zero-phonon ion resonance energy (*cf.* E_{PIR} in figure S1a) and $\lambda_{\pm}$ is proportional to the electron-phonon coupling strength η:

$$\lambda = \int_0^{\infty} J(\Omega)\Omega \, \mathrm{d}\Omega = \frac{\eta}{2}\left(\Omega_{\mathrm{max}}^2 - \Omega_{\mathrm{min}}^2\right) \tag{S3}$$

Fitting was performed with a voltage scale of 0.9 to account for voltage drop of $\alpha_{\mathrm{P}} = 10\%$ across the NaCl. In Fig. S1 the x-axis of the fit data is rescaled to match the experimental data.

TABLE I. **Fitted parameters obtained from polaron model ($\alpha_{\mathrm{P}} = 10\%$).**

**Parameter**	$\mathbf{TBP}_{111}$	$\mathbf{Pc}_{111}$	$\mathbf{TBP}_{110}$
$E_{D_0^+}$(meV)	1812 ± 14	1984 ± 5	2248 ± 5
λ_+(meV)	268 ± 11	216 ± 6	303 ± 7
$E_{D_0^-}$(meV)	937 ± 8	422 ± 6	544 ± 2
λ_-(meV)	310 ± 11	311 ± 7	297 ± 3

VOLTAGE DEPENDENT STML INTENSITES

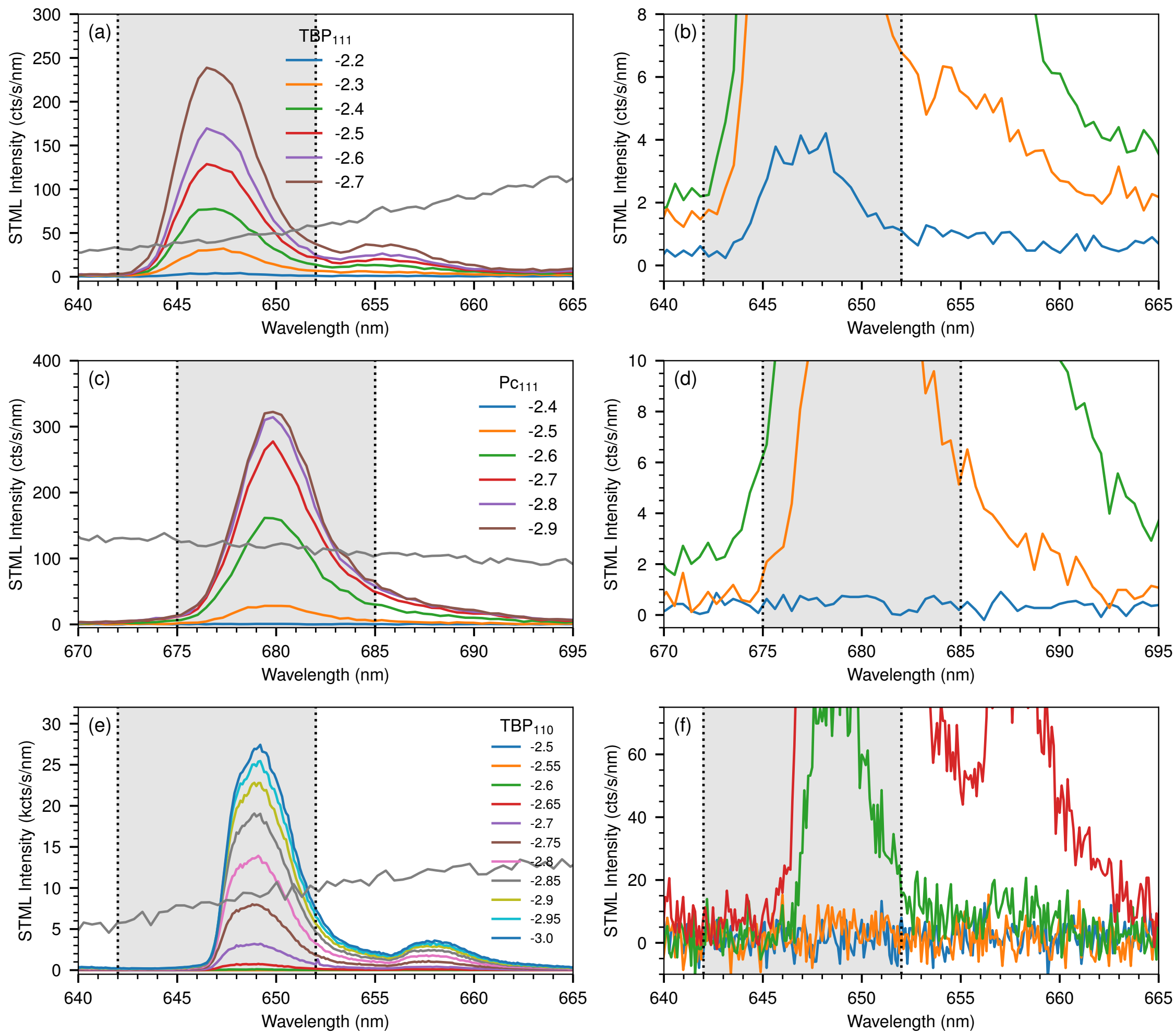

FIG. S2. STML spectra taken on H_2TBP/NaCl/Ag(111), H_2Pc/NaCl/Ag(111) and H_2TBP/NaCl/Ag(110). The right column shows a zoom in onto the low intensity spectra taken at low bias voltages. The gray area marks the integration area and the grey line represents the reference plasmon spectrum in arbitrary units.

RATE EQUATION MODEL FOR VOLTAGE DEPENDENT EMISSION

The voltage dependence of the emission rate was simulated with a simple rate equation model, similar to the one discussed in Ref. 4. Here we consider only three states (see Fig. 2a of the main text), namely S_0, S_1 and D_0^+. The energy $E_{S_1} = 1.915\,\mathrm{eV}$ of the excitonic state is given by the experimentally measured photon energy of the primary emission line and from the polaron fit we extracted the energy $E_{D_0^+}$

The voltage dependent transition rates between the neutral and the charged state are given by integration of the polaron spectral profile as described in equation (S1):

$$\Gamma^{t/s}_{S_i \to D_0^+}(V_{\mathrm{eff}}) = \frac{2\hat{V}_{t/s}}{h} \int_{V_{\mathrm{eff}}}^{\infty} S(E_{D_0^+} - E_{S_i}, +1, V')\ \mathrm{d}V'$$

$$\Gamma^{t/s}_{D_0^+ \to S_i}(V_{\mathrm{eff}}) = \frac{\hat{V}_{t/s}}{h} \int_{-\infty}^{V_{\mathrm{eff}}} S(E_{S_i} - E_{D_0^+}, -1, V')\ \mathrm{d}V',$$

with $\hat{V}_{t/s}$ being the coupling strength of the molecule to tip and surface. The effective bias voltage V_{eff} between molecule and corresponding lead is given by the voltage drop $\alpha_{\mathrm{RE}} \approx 0.1$:

$$V^t_{\mathrm{eff}} = (1 - \alpha_{\mathrm{RE}})V_b \tag{S4}$$

$$V^s_{\mathrm{eff}} = -\alpha_{\mathrm{RE}} V_b. \tag{S5}$$

The additional factor of two for the singlet to doublet transition $S_i \to D_0^+$ yields from the multiplicities of the involved states and is given by the Clebsch-Gordan coefficients Υ_{fi}. As an approximation, we assume the relaxation energy λ to be the same for all transitions between the positive ion resonance D_0^+ and the charge neutral states S_0 and S_1:

$$\lambda = \lambda^0_+ = \lambda^0_0 = \lambda^1_+ = \lambda^1_0 \tag{S6}$$

In the bias range of interest we further assume some transition rates to be constant:

$$\Gamma^s_{S_0 \to D_0^+} = 0 \tag{S7}$$

$$\Gamma^s_{D_0^+ \to S_0} = 2.5\hat{V}_s/h \tag{S8}$$

$$\Gamma^t_{D_0^+ \to S_0} = \Gamma^t_{D_0^+ \to S_1} = 0 \tag{S9}$$

$$\Gamma^t_{S_1 \to D_0^+} = 2\hat{V}_t/h \tag{S10}$$

We estimated the coupling of the molecule to the substrate to be $\hat{V}_s = 4\,\mu\mathrm{eV}$ (see below) for all orbitals and used the coupling to the tip $\hat{V}_t$ as a fitting parameter. In this simplified

model we do not directly include T_1, since experimentally there is no indication of triplet pumping [4]. However, the presence of the triplet state causes an increase in the dark current as it opens another channel to neutralize the molecule via sample from the D_0^+ state into T_1 and then decay non-radiatively back into the ground state S_0. To capture this effect, the transition rate $\Gamma^s_{D_0^+\to S_0}$ is multiplied by a factor of 2.5, which is the sum of the Clebsh-Gordan coefficients $\Upsilon_{D_0^+\to S_0} = 1$ and $\Upsilon_{D_0^+\to T_1} = 1.5$. For the radiative emission $\Gamma^{\mathrm{rad}}_{S_1\to S_0} = \hat{V}_{\mathrm{rad}}/h$ the lifetime was estimated from the width of the emission peak to be in the order of $\hat{V}_{\mathrm{rad}} = 1\,\mathrm{meV}$.

The occupation probability N_i of the three considered states S_0, S_1 and D_0^+ is calculated using a differential rate equation for the steady state condition

$$0 = \sum_j \Gamma_{j\to i}(V_b)N_j - \Gamma_{i\to j}(V_b)N_i \tag{S11}$$

for given bias voltage V_b. The net current flowing through the molecule is then defined by

$$I_{\mathrm{mol}}(V) = -e^-(\Gamma^t_{S_0\to D_0^+}N_{S_0} + \Gamma^t_{S_1\to D_0^+}N_{S_1}). \tag{S12}$$

The number of photons emitted is given by the probability of occupation of S_1 and the radiative emission rate:

$$J^0_{\mathrm{ph}}(V) = \Gamma^{\mathrm{rad}}_{S_1\to S_0}N_{S_1}. \tag{S13}$$

To account for the experimental detection efficiency, a prefactor $0 < A < 1$ is introduced to obtain the experimental photon counts:

$$J_{\mathrm{ph}}(V) = A\ J^0_{\mathrm{ph}}(V) = A\ \Gamma^{\mathrm{rad}}_{S_1\to S_0}N_{S_1} \tag{S14}$$

with an expectation value of about $A \sim 10^{-4}$ [5].

The modeled current and light intensity are fitted to the experimental data, using tip coupling $\hat{V}_t$, voltage drop α_{RE} and photon detection efficiency A as fit parameters. The resulting fits are shown in Fig. S3 and the corresponding parameter listed in table II.

It should be noted that the calculated STML intensity for TBP_{111} is very sensitive to the voltage drop α_{RE}, because the system is right at the onset regime of emission and slight changes in α_{RE} cause an exponential increase or decrease in emission intensity. Accordingly, the obtained error value for α_{RE} from the fit is less than $1\cdot 10^{-4}$ percent points. However, the value of α_{RE} itself also depends on the voltage drop α_{P} used for the polaron fit, effectively increasing the actual error margin of α_{RE} (see section below).

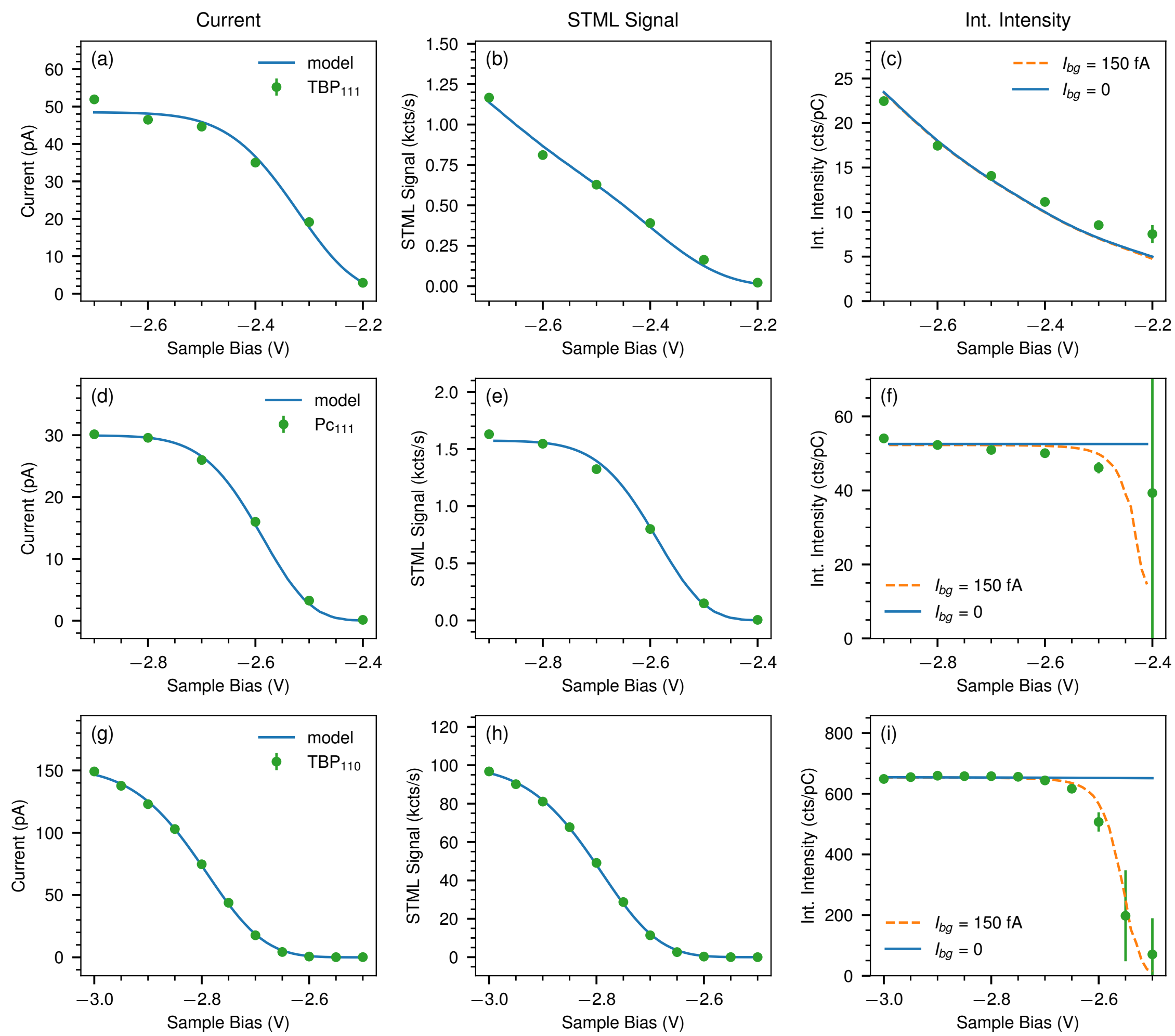

FIG. S3. **Fitting of STML data.** Bias dependency of the experimental current and STML intensity of the dominant emission line, as well as the current normalized integrated intensity for TBP_{111} (a-c), Pc_{111} (d-f) and TBP_{110} (e-h). The blue solid lines are fit of the rate equation model to the experimental data, and the orange dashed line represents current normalized STML intensity, assuming the presence of an additional background current of 150 fA.

For the normalized STML intensity one also needs to consider the effect of an additional background current flowing directly between the tip and the underlying substrate. The total current is given by the sum of the current through the molecule (I_{mol}) and the additional background current (I_{bg}):

$$I_{\mathrm{tot}} = I_{\mathrm{mol}} + I_{\mathrm{bg}}, \tag{S15}$$

with I_{tot} corresponding to the current measured in the experiment. In particular, at the onset of the PIR, these two currents become comparable in magnitude ($I_{\mathrm{mol}} \approx I_{\mathrm{bg}}$). In

especially in the case of TBP_{111} and Pc_{111} this causes a drop in the normalized emission intensity

$$j_{\text{ph}} = \frac{J_{\text{ph}}}{I_{\text{tot}}} = \frac{J_{\text{ph}}}{I_{\text{mol}} + I_{\text{bg}}}, \tag{S16}$$

although $J_{\text{ph}}/I_{\text{mol}}$ is expected to be constant in the bias range of interest. To account for this effect in the simulation, we added an additional background current of $I_{\text{bg}} = 150\,\text{fA}$ to the calculated current I_{mol} to reproduce the drop in j_{opt}, as shown in Fig. S3.

TABLE II. **Parameters used to model the STML intensity.** $E_{D_0^+}$ and λ^+ were obtained from the polaron model using $\alpha_{\text{P}} = 10\%$. A, α_{RE} and $\hat{V}_t$ were used as fitting parameters for the rate equation model.

**Parameter**	$\textbf{TBP}_{111}$	$\textbf{Pc}_{111}$	$\textbf{TBP}_{110}$
E_{S_0} (eV)		0	
E_{S_1} (eV)	1.915	1.810	1.915
$E_{D_0^+}$ (eV)	1.812	2.123	2.248
λ^+ (meV)	268	186	303
A ($\times 10^{-4}$)	1.08 ± 0.04	0.30 ± 0.01	3.70 ± 0.03
α_{RE} (%)	10.4	10.8 ± 0.2	9.0 ± 0.1
$\hat{V}_t$ (μeV)	0.70 ± 0.02	0.41 ± 0.01	2.73 ± 0.03
$\hat{V}_s$ (μeV)		4	

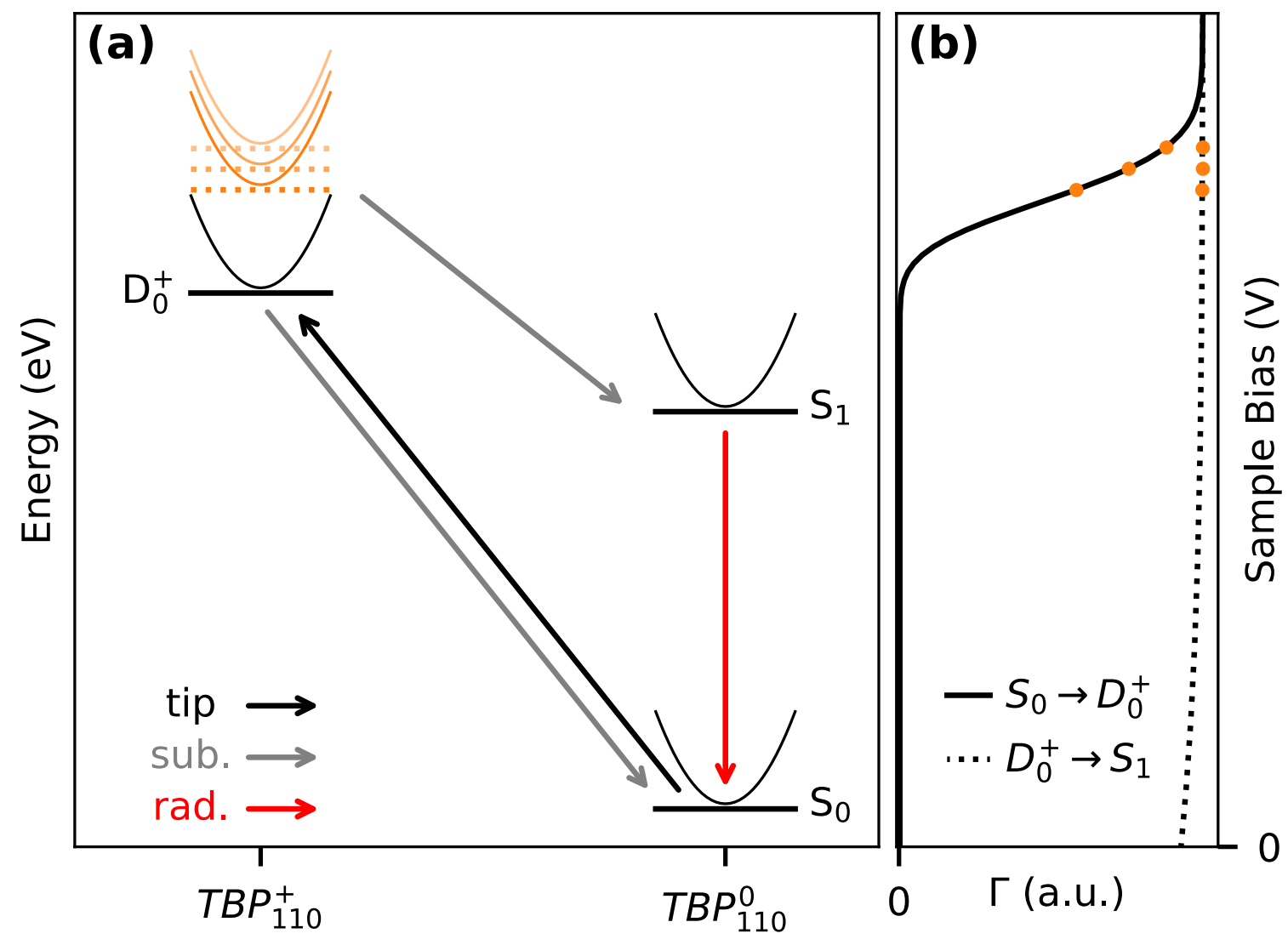

FIG. S4. (a) Many-body diagram describing the electronic states and transitions of a TBP_{110} molecule during STML experiments, analogous to Fig. 2a,b in the main text. Black and grey arrows connecting different TBP_{110} charge states represent electron transfer involving the tip and substrate while the red arrow represents radiative relaxation. The excess relaxation energy needed for charge transport is depicted as a parabola above each state. A voltage dependent shift in D_0^+ energy, $\Delta E(V)$, is present during experiments and indicated by a series of orange dashed lines. (b) Voltage dependent $S_0 \rightarrow D_0^+$ and $D_0^+ \rightarrow S_1$ transition rates. Orange circles correlate with $\Delta E(V)$ shown in (a).

ROLE OF VOLTAGE DROP IN SIMULATION

The voltage drop over the NaCl layer is a crucial parameter to model the system and simulate the light emission from the junction. In the following we will be discussing the effect of different assumed voltage drops α_P in the polaron fit.

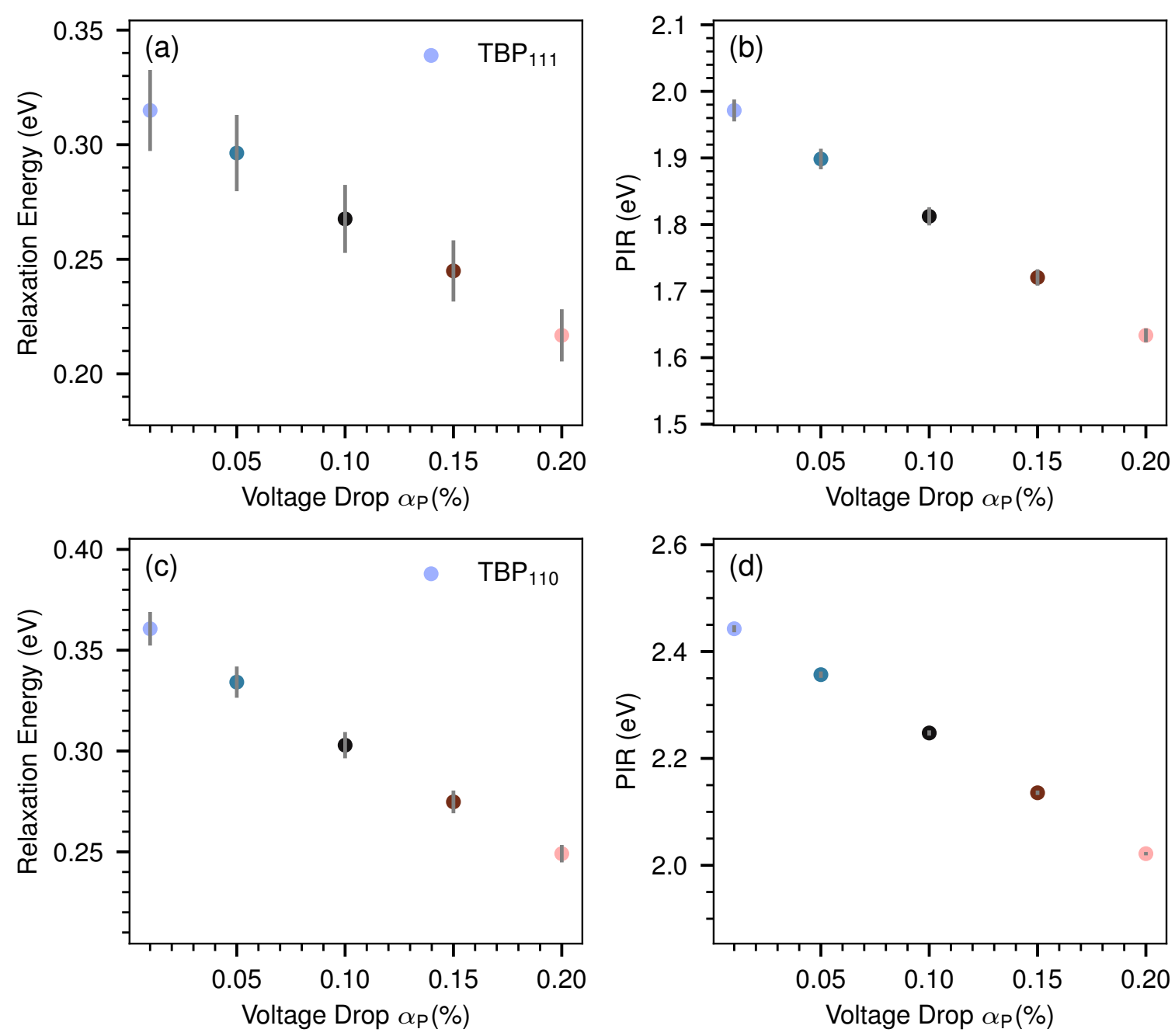

FIG. S5. Dependency of relaxation energy λ^+ and ion resonance energy $E_{D_0^+}$ on assumed voltage drop α_P, obtained by polaron fit.

We fitted the experimental $\mathrm{d}I/\mathrm{d}V$ spectra assuming five different values for α_P, ranging between 1% and 20%. The obtained best fit parameters λ^+ and $E_{D_0^+}$ for each value of α_P are shown in Fig. S5 for both TBP_{111} (top row) and TBP_{110} (bottom row). With increasing voltage drop, we observe an almost linear reduction in relaxation energy λ^+, as well as in the ion resonance energy $E_{D_0^+}$, with good fits to experiment in all cases. In the strong electron-phonon coupling regime, with relaxation energies much larger than the phonon energies ($\lambda^+ \gg \hbar\Omega$), an increase (decrease) in relaxation energy will linearly widen (narrow) the ion resonance peak and shift it to higher (lower) energies, while maintaining the approximate gaussian line shape. For this reason, the experimental spectra can be fitted well with any voltage drop in the voltage drop range considered here.

In the next step, the obtained values for the relaxation and ion resonance energy were used in the rate equation model, to reproduce the experimental data sets as described in the

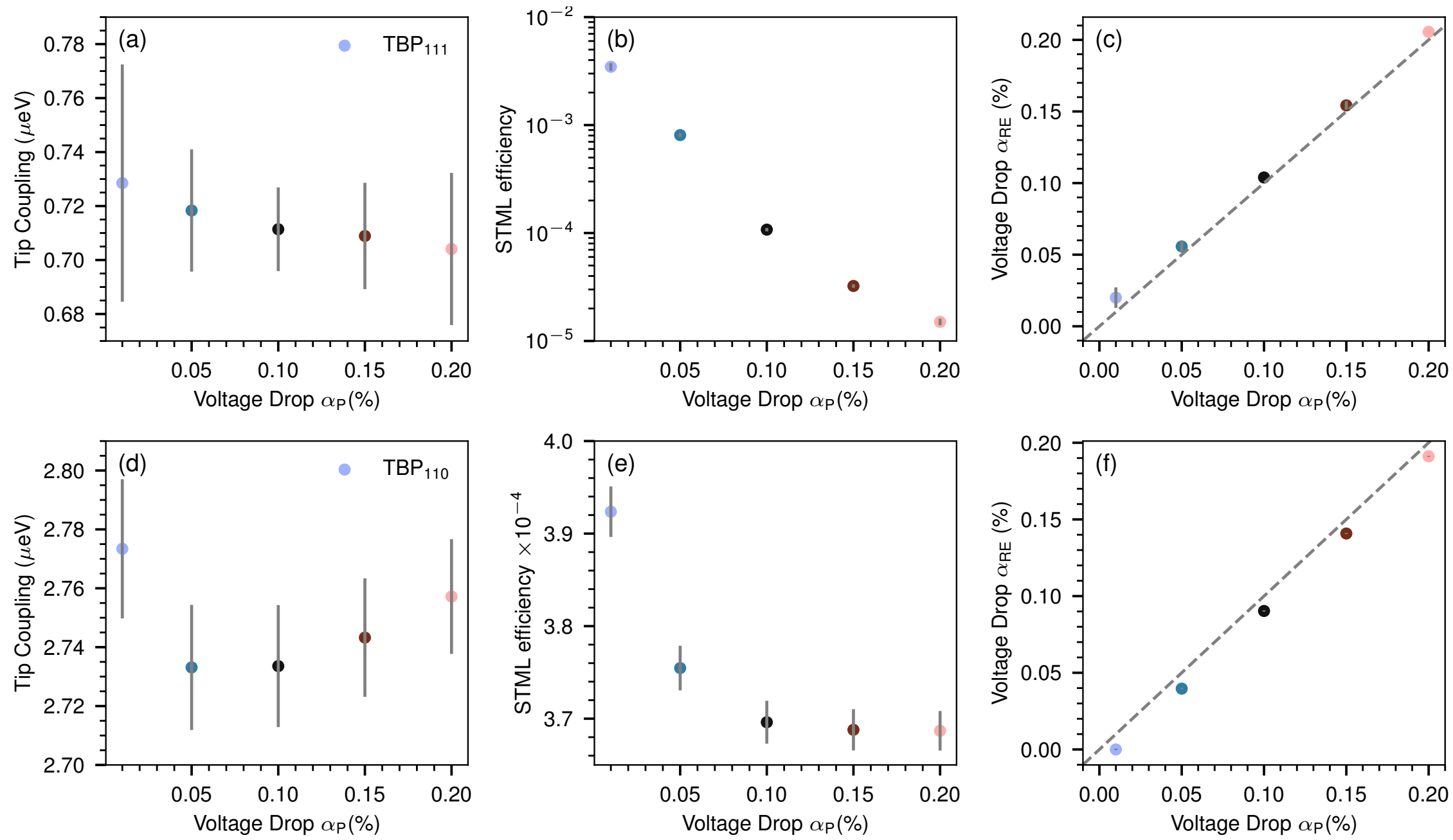

FIG. S6. Dependency of tip coupling strength $\hat{V}_t$, STML efficiency A and rate equation voltage drop α_{RE} on the assumed polaron voltage drop α_{P}, obtained by rate equation fit.

section above. Fig. S6 shows the best fit parameters in dependence of the polaron voltage drop α_{P}, again for TBP_{111} and TBP_{110}. In both cases, the tip coupling strength does not depend strongly on α_{P}, which to be expected, as this parameter mainly scales the amplitude of the current. On the other hand, the STML efficiency A depends almost exponentially on α_{P} for TBP_{111}, spanning several orders of magnitude. In contrast, it is nearly constant for TBP_{110}. This effect can be rationalized, by a closer look to the voltage dependent transition rates $\Gamma^t_{S_0\to D_0^+}$ and $\Gamma^s_{D_0^+\to S_1}$ for different voltage drops, as depicted in Fig. S7. The tip mediated rate $\Gamma^t_{S_0\to D_0^+}$ is the same for all α_{P} (Fig. S7a,c), since it represents the main contribution to the tunneling current and is accordingly fitted to experiment. In contrast, the sample mediated rate $\Gamma^s_{D_0^+\to S_1}$ depends strongly on the initially assumed voltage drop (Fig. S7b). The gray areas indicate the voltage range of the experimental data set. For TBP_{111} the voltage range is just at the exponential onset of the transition rate, in especially for small values of α_{P}. Accordingly, $\Gamma^s_{D_0^+\to S_1}$ increases exponentially with increasing α_{P} for a given voltage in that range. In order to fit the simulated light emission $J_{\mathrm{ph}} = A\ J^0_{\mathrm{ph}}$ to experimental data, the STML efficiency A has to decrease exponentially, as we observed. For TBP_{110} the voltage range (gray area in Fig. S7d) is already at the plateau of $\Gamma^s_{D_0^+\to S_1}$,

hence the near constant values of A.

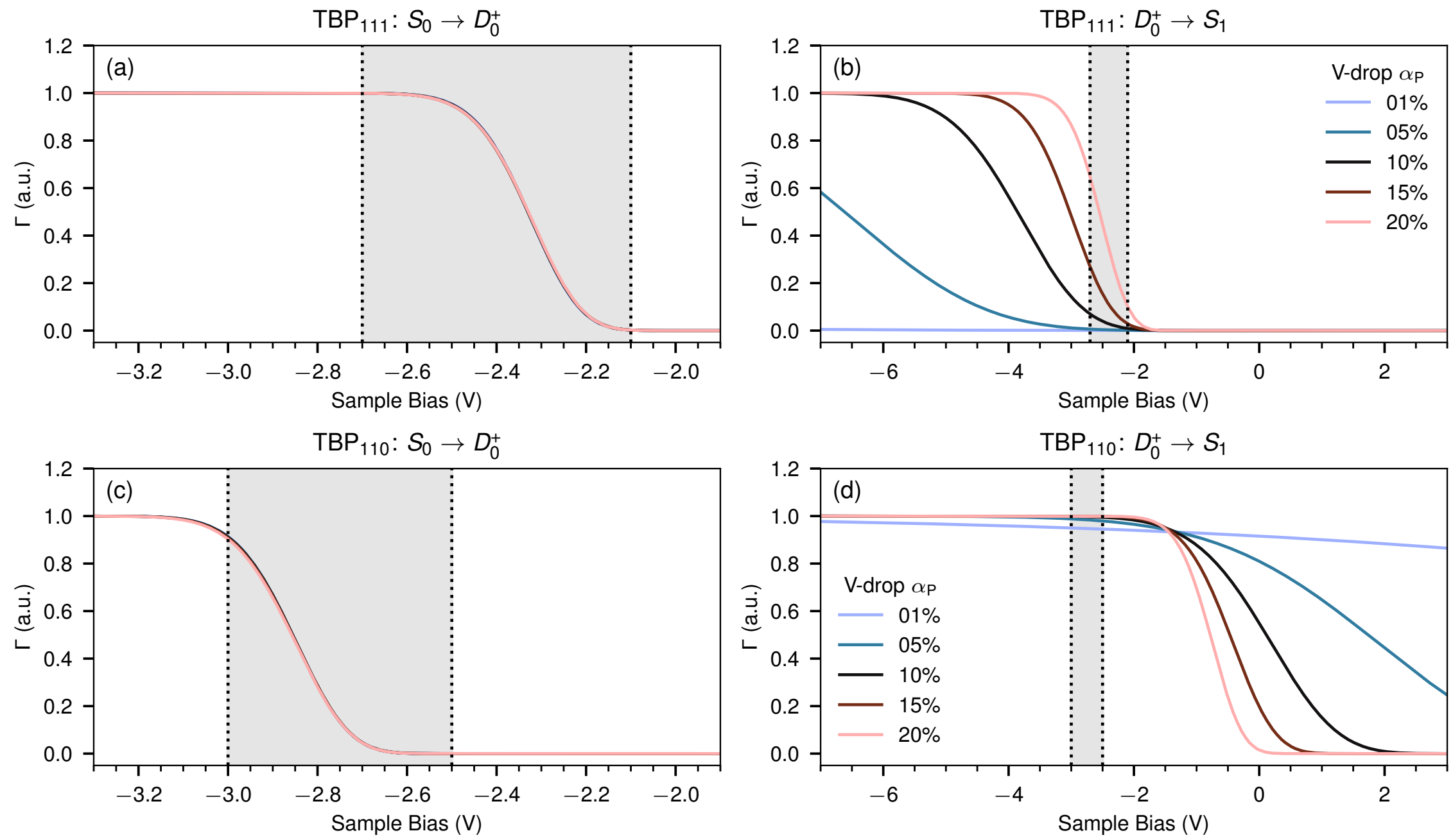

FIG. S7. Dependency of the transition rates on the assumed polaron voltage drop α_{P}, obtained by rate equation fit. The gray areas mark the voltage range of the experiment.

The obtained values for α_{RE} are shown in Fig. S6c,f. As expected, they show a near linear behavior, with α_{RE} being within one percent point around α_{P}. The slight deviation of the two voltage drop parameters are reasoned by the tip shape and position dependency of the voltage drop, which can lead to slight difference between data sets.

The effect of the assumed voltage drop on the simulated STM current and STML intensity is shown in Fig. S8 for TBP_{111} and Fig. S9 for TBP_{110}. In the first case, the STML intensity J_{ph} can be fitted decently for most voltage drops, except for $\alpha_{\mathrm{P}} = 20\%$, which saturates too fast and cannot capture the experimental behavior at higher bias voltages. The deviation of J_{ph} for $\alpha_{\mathrm{P}} = 1\%$ is caused by a shoulder in the transition rate $\Gamma^{s}_{D_0^+ \to S_1}$, due to discernable vibronic states on the low energy flank of the polaron peak (see Fig. S8e). These shoulders depend strongly on the electron-phonon coupling function $J(\Omega)$ (see above) and are amplified by the very small voltage of 1%, but should not be considered an exact modeling of the transition rate, since $J(\Omega)$ is approximated by a simple rectangular function. Nevertheless, we can conclude from these fits that the voltage drop for TBP_{111} has to be smaller than 20% to fit with the experiment. Another estimate can be done by consideration of the STML

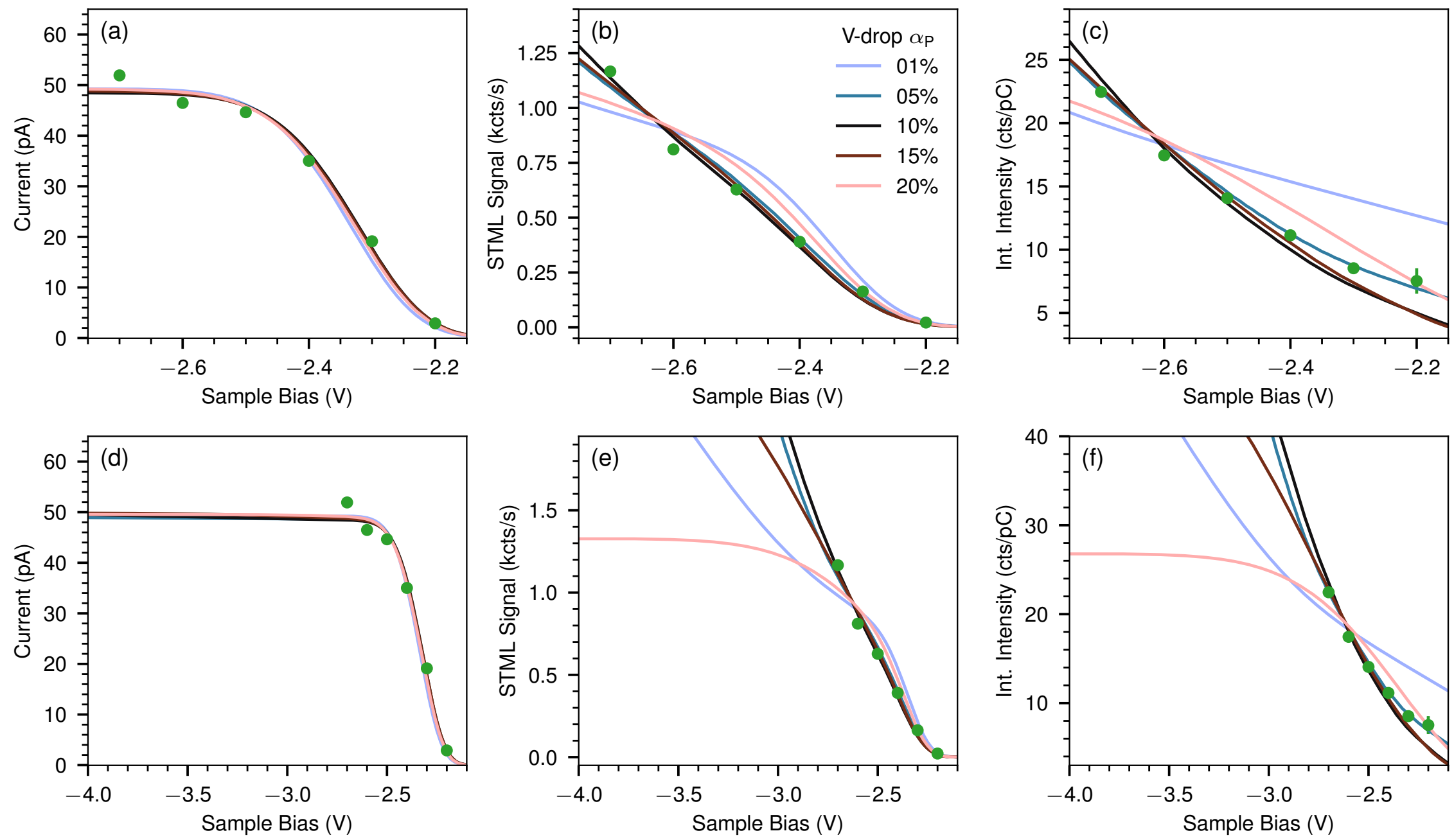

FIG. S8. STML fits for TBP_{111} for several assumed polaron voltage drop α_P. In the bottom row, the simulated curves are extrapolated to higher absolute bias voltages.

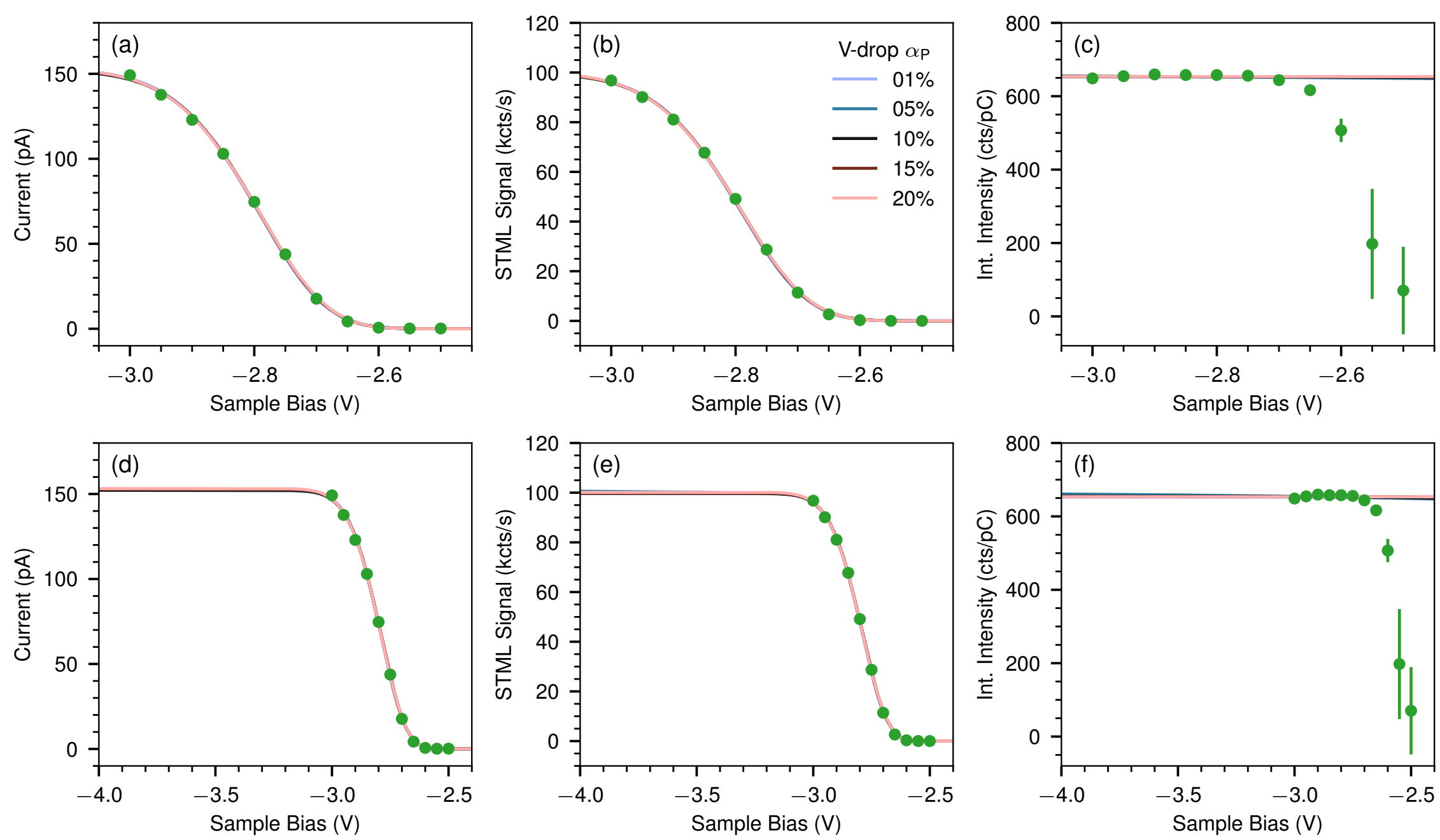

FIG. S9. STML fits for TBP_{110} for several assumed polaron voltage drop α_P. In the bottom row, the simulated curves are extrapolated to higher absolute bias voltages.

efficiency A. A typical estimation of the STML efficiency is $A \sim 10^{-4}$ [5], which would match with a voltage drop between 5% and 15% (see Fig. S6b).

For TBP_{110}, the assumed voltage drop has little effect on the STML fit, therefore we cannot deduce how strong the voltage drop is in the experiment. Since the transition $\Gamma^{s}_{D_0^+ \to S_1}$ is always fully allowed, the STML efficiency parameter A is nearly constant as well, with $A \sim 4 \cdot 10^{-4}$.

In accordance with literature, we decided to assume a voltage drop of 10% for both substrates. The experimental value is expected to be close within a few percent points and the precise value of α does not change significantly the observed voltage dependent behavior of the STML intensity.

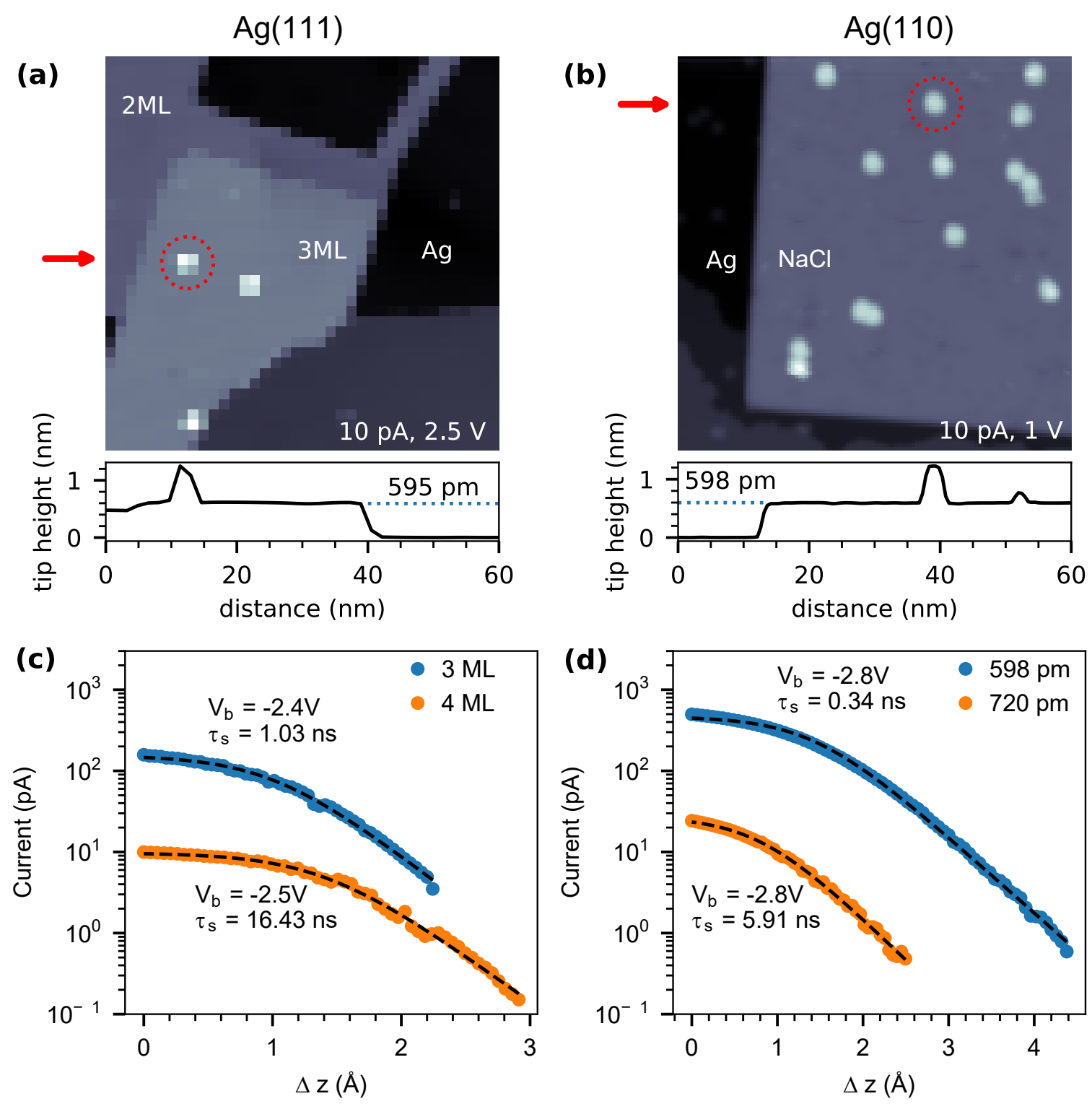

FIG. S10. Overview images of H_2TBP molecules adsorbed on few-layer NaCl films of comparable topographic heights grown on (a) Ag(111) and (b) Ag(110). STM topographic height profiles are shown along the bottom axis of the images with line profile locations indicated by red arrows. (c) I(z) approach curves measured on H_2TBP molecules adsorbed on 3 ML NaCl (blue dots) and 4 ML NaCl (orange dots) grown on Ag(111). The 3 ML curve is measured on the molecule highlighted with a red dashed circle. (d) I(z) approach curves measured on H_2TBP molecules adsorbed on NaCl islands grown on Ag(110) with apparent heights comparable to those shown in (c). Black dashed lines show fits to the I(z) data which were used to extract charge state lifetimes, τ_s, indicated in the figures. Each measurement was recorded with a sample bias, V_b, shown alongside the corresponding I(z) curve. The molecule highlighted in (a) was used to record the STML map shown in Fig. 4a while the highlighted molecule in (b) was used in Fig. 3c.

NACL DECOUPLING ON AG(111) AND AG(110)

The charge state lifetime of H_2TBP on the different NaCl layers was determined by fitting a simple rate model to $I(z)$ curves, using the function [6]:

$$I(z) = \frac{e^-}{\tau_s + \tau_t^0 \; e^{2\kappa\Delta z}}, \quad \text{(S17)}$$

with e^- being the elementary charge, $\tau_s = 1/\Gamma_s$ the lifetime of the charge state, $\tau_t^0 = 1/\Gamma_t^0$ the inverse tunneling rate from tip to molecule at relative tip height $\Delta z = 0$ and κ being the decay constant.

SIMULATION OF FLUORESCENCE MAPS

The hyper-resolved flourescence maps were simulated using the rate equation and polaron model described above. Here, we consider tunneling into the HOMO and HOMO-1 as possible excitation pathways. The spatial dependence of the tip–molecule coupling strength $\hat{V}_t$ is obtained from DFT calculations by extrapolating the single-particle wave function $\Psi_i(\mathbf{r})$ of molecular orbital i into the vacuum region:

$$\hat{V}_t^i(\mathbf{r_t}) = \hat{V}_t^0 \frac{|\Psi_i(\mathbf{r_t})|^2}{\max\left(|\Psi_i(\mathbf{r_t})|^2\right)}, \tag{S18}$$

where $\hat{V}_t^0$ denotes the maximum tip–molecule coupling strength, corresponding to the value obtained from fits with the rate-equation model. Figure S11a,b show the spatial dependence of the tip coupling for the HOMO and HOMO–1, respectively, extrapolated to a tip height of 10 Å.

For the effective tip–molecule coupling, we consider tunneling contributions into both the HOMO and the HOMO-1:

$$\hat{V}_t(\mathbf{r_t}) = \hat{V}_t^{\mathrm{HOMO}}(\mathbf{r_t}) + \beta\, \hat{V}_t^{\mathrm{HOMO-1}}(\mathbf{r_t}) \tag{S19}$$

Excitation via the HOMO–1 orbital would populate the higher-energy state D_1^+. However, due to the long lifetime of the charged state (on the order of several hundred picoseconds), we can assume that the molecule relaxes to D_0^+ before neutralizing into S_1. The mixing ratio β depends on the experimental bias voltage. In the simulations, we use $\beta_{111} = 1.0$ for TBP_{111} and $\beta_{110} = 0.4$ for TBP_{110}. The comparison between experimental and simulated current maps is shown in Figure S12

The tip-position dependence of the voltage drop is modeled using a two-point-charge geometry as depicted in figure S11c. The electric potential in the junction, induced by the applied bias voltage V_b is approximated as $\phi_b(\mathbf{r}) = \varphi(\mathbf{r})V_b$, where

$$\varphi(\mathbf{r}) = 1 - \left(\frac{R^2}{2z_t} + R\right)\left[\frac{1}{\sqrt{x^2+y^2+(z-z_p)^2}} - \frac{1}{\sqrt{x^2+y^2+(z+z_p)^2}}\right], \tag{S20}$$

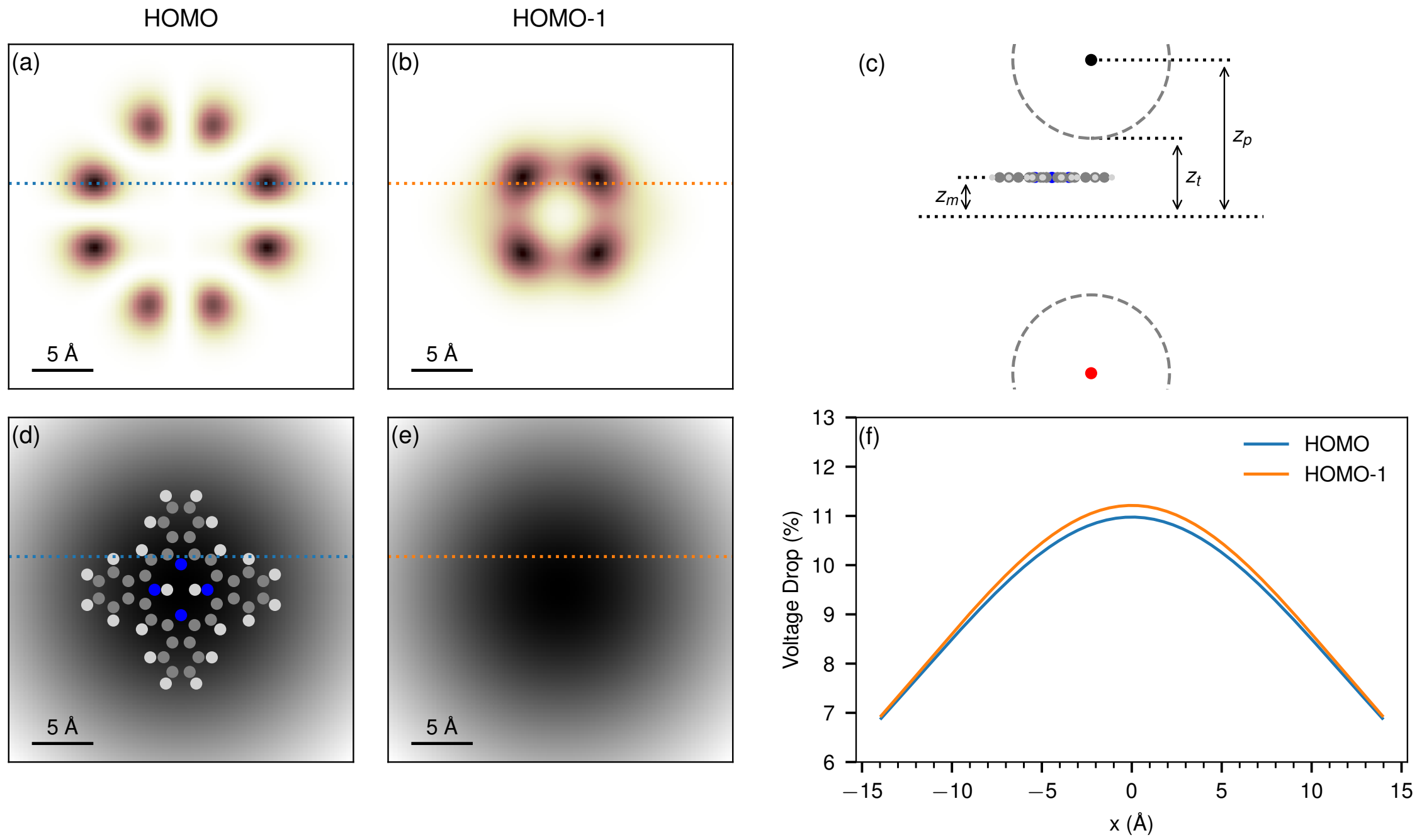

FIG. S11. H_2TBP in the STM junction. (a,b) Spatial dependence of the tip–molecule coupling to the HOMO and the HOMO-1, respectively, for a tip–molecule distance of 10 Å. (c) Schematic of the junction model, represented by two point charges of opposite sign. The molecule is positioned at a height z_m above the surface plane and the tip (grey dashed line) is assumed to have a radius of $R = z_p - z_t$. (d,e) Spatial dependence of the effective voltage drop for the HOMO and the HOMO-1, respectively. (f) Line cuts along the dashed lines in (a,b) and (d,e), showing the effective voltage drop α.

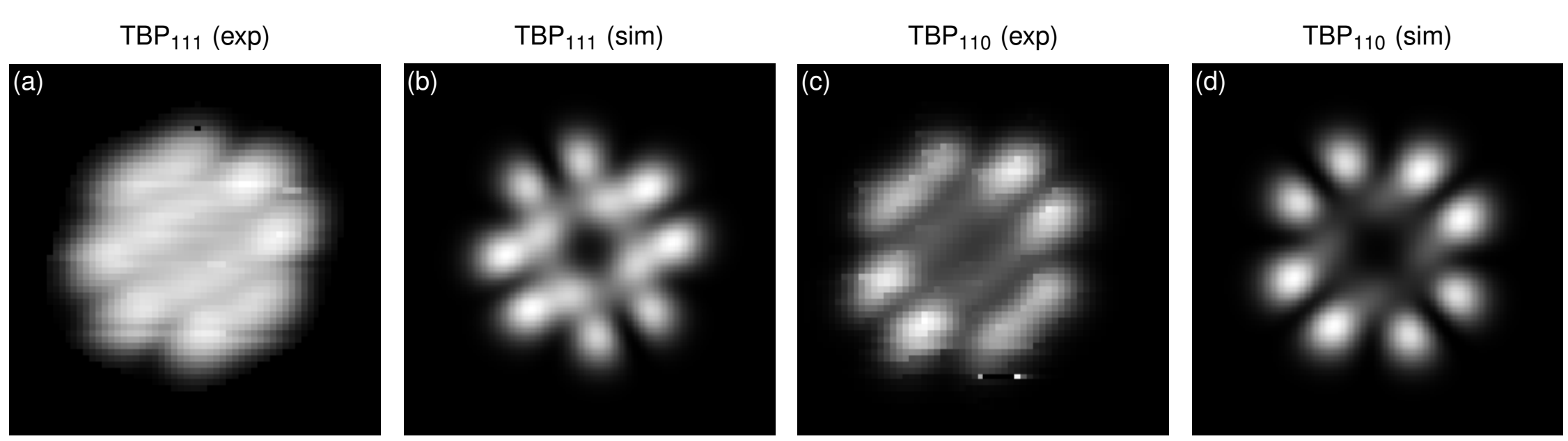

FIG. S12. Experimental current maps, extracted from the spatially resolved fluorescence maps, and spatial dependency of the tip coupling strength, according to eq. (S19), for TBP_{111} (a,b) and TBP_{110} (c,d).

with $R = z_p - z_t$ denoting the effective tip radius [7]. The effective potential applied to the molecule is then given by

$$V_m(\mathbf{r_t}) = V_b \int \mathrm{d}\mathbf{r}\Psi^*(\mathbf{r})\varphi(\mathbf{r}-\mathbf{r_t})\Psi(\mathbf{r}), \tag{S21}$$

with Ψ representing the wave function of the corresponding Dyson orbital. In good approximation, we use the single-particle wave functions of H_2TBP as Ψ.

Finally, the effective voltage drop α is defined as the ratio between the applied bias V_b and the effective potential V_m applied to the molecule:

$$\alpha(\mathbf{r}) = 1 - \frac{V_m}{V_b} = 1 - \int \mathrm{d}\mathbf{r}\Psi^*(\mathbf{r})\varphi(\mathbf{r}-\mathbf{r_t})\Psi(\mathbf{r}). \tag{S22}$$

The spatial dependence of the voltage drop for the HOMO and the HOMO-1 is shown in Figure S11d,e for two point charges, located at $z_p = \pm 20\,\text{Å}$, and a vacuum gap of $z_t = 10\,\text{Å}$. The molecule is displaced by $z_m = 1\,\text{Å}$ from the surface plane to account for the dielectric decoupling layer. The junction geometry was chosen such that the voltage drop reaches $\alpha \approx 10\,\%$ at the position of maximum tip–molecule coupling for the HOMO orbital. Figure S11f shows two line cuts of the simulated voltage drop, illustrating that the voltage drop is largest when the tip is directly above the molecule and decreases with increasing lateral distance.

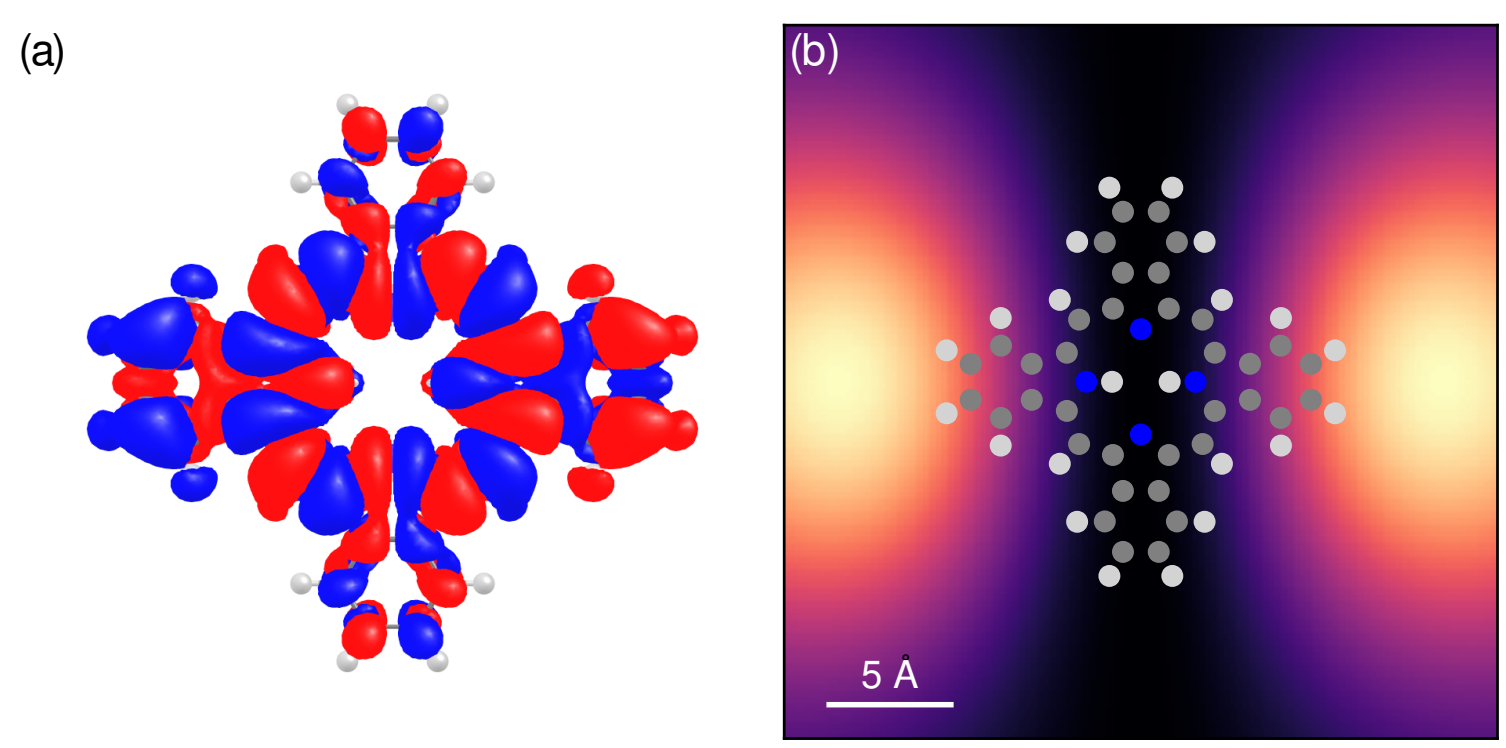

FIG. S13. Transition density of the S_1 state of H_2TBP. (a) iso-surface of the transition density mapped onto stick-and-ball model of H_2TBP. (b) Radiative coupling strength $\hat{V}_{\mathrm{rad}}(\mathbf{r_t})$ according to eq. (S23).

The tip plasmon is modeled using the same two-point-charge geometry described above. The coupling strength to the transition density ρ_{if} between states i to f is then given by [8]:

$$\hat{V}_{\mathrm{rad}}(\mathbf{r_t}) = \hat{V}^0_{\mathrm{rad}} \frac{|g(\mathbf{r_t})|^2}{\max\left(|g(\mathbf{r_t})|^2\right)}, \tag{S23}$$

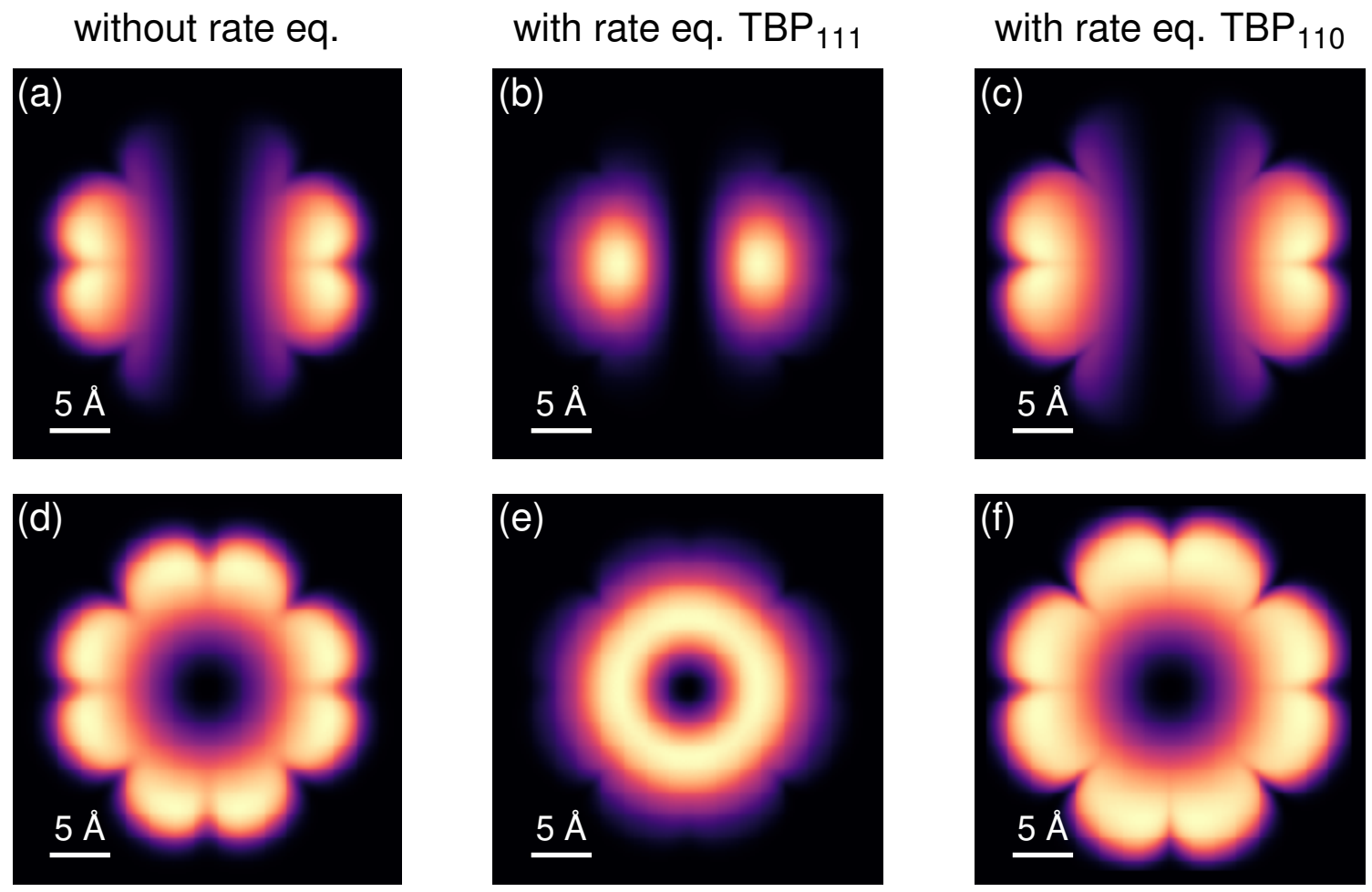

FIG. S14. Simulated flourescence maps for H_2TBP. (a) Map obtained using the simple model (Eq. S26), without the rate equation model or the effects of voltage drop. (b,c) Fluorescence maps calculated using the rate-equation model, including a spatially dependent voltage drop, for TBP_{111} and TBP_{110}, respectively. (d-f) Fluorescence maps including tautomerization effects, constructed by rotating each map in (a–c) by 90° and summing the resulting pair.

with $\hat{V}^0_{\mathrm{rad}} = 1\,\mathrm{meV}$ and

$$g(\mathbf{r_t}) = \int \mathrm{d}\mathbf{r} \rho_{ij}(\mathbf{r})\phi(\mathbf{r}-\mathbf{r_t}). \tag{S24}$$

The tip potential $\phi(\mathbf{r})$ is described by the electrostatic potential of the two point charges as depicted in Figure S11e:

$$\phi(\mathbf{r}) = \frac{1}{\sqrt{x^2+y^2+(z-z_p)^2}} - \frac{1}{\sqrt{x^2+y^2+(z+z_p)^2}}. \tag{S25}$$

Figure S13 shows the transition density ρ_{if} for the S_1 state of H_2TBP, together with the corresponding spatial map of the plasmon coupling strength $\hat{V}_{\mathrm{rad}}(\mathbf{r_t})$.

For systems that are not affected by spatially dependent gating, normalized STML maps can be reproduced by multiplying the tip-plasmon coupling strength $|g(\mathbf{r_t})|^2$ with the tip coupling rate $\hat{V}_t(\mathbf{r_t})$ and subsequent renormalization, while including an additional background current [9]:

$$j^{\mathrm{sim}}_{\mathrm{ph}}(\mathbf{r}) \propto |g(\mathbf{r_t})|^2 \, \frac{\hat{V}_t(\mathbf{r_t})}{\hat{V}_t(\mathbf{r_t}) + \gamma_{\mathrm{bg}}}, \tag{S26}$$

with $\gamma_{\mathrm{bg}} = 0.01\hat{V}^0_t$. The resulting map is shown in Figure S14a for the S_1 state of H_2TBP. Figure S14d shows the corresponding map, when fast tautomerization is assumed, with an equal weighting of the two tautomers.

To capture the effect of spatially dependent voltage drop, we simulate the STML maps by solving the rate equation at each tip position, using the definitions for $\hat{V}_t(\mathbf{r})$, $\hat{V}_{\mathrm{rad}}(\mathbf{r})$, and $\alpha(\mathbf{r})$ introduced above. The resulting fluorescence map is then normalized by the current, including a dark background current of $I_{\mathrm{bg}} = 150\,\mathrm{fA}$:

$$j_{\mathrm{ph}}^{\mathrm{RE}}(\mathbf{r}) = \frac{J_{\mathrm{ph}}(\mathbf{r})}{I_{\mathrm{mol}}(\mathbf{r}) + I_{\mathrm{bg}}}. \tag{S27}$$

Figure S14b,e and S14c,f show the simulated STML maps for TBP_{111} and TBP_{110}, respectively, both without and with the inclusion of tautomerization. The simulated maps for TBP_{110} are nearly identical to those obtained from the simplified model shown in Figure S14a,d, supporting the conclusion that TBP_{110} is largely insensitive to the gating effect. In contrast, TBP_{111} deviates from the idealized model, exhibiting a ring-shaped intensity distribution with the maximum shifted towards the center of the molecule. This result is consistent with the experimental maps (Figure 4) and highlights that the inclusion of the gating effect introduces an additional layer of complexity in interpretation of spatially resolved STML data.

* eve.ammerman@empa.ch

[1] J. Repp, G. Meyer, S. Paavilainen, F. E. Olsson, and M. Persson, Phys. Rev. Lett. **95**, 225503 (2005).

[2] S. Fatayer, B. Schuler, W. Steurer, I. Scivetti, J. Repp, L. Gross, M. Persson, and G. Meyer, Nat. Nanotechnol. **13**, 376 (2018).

[3] K. Vasilev, S. Canola, F. Scheurer, A. Boeglin, F. Lotthammer, F. Chérioux, T. Neuman, and G. Schull, ACS Nano **18**, 28052 (2024).

[4] K. Kaiser, A. Rosławska, M. Romeo, F. Scheurer, T. c. v. Neuman, and G. Schull, Phys. Rev. X **15**, 021072 (2025).

[5] K. Kuhnke, C. Große, P. Merino, and K. Kern, Chemical Reviews **117**, 5174–5222 (2017).

[6] K. Kaiser, L.-A. Lieske, J. Repp, and L. Gross, Nature Communications **14** (2023), 10.1038/s41467-023-40692-1.

[7] N. Krane, C. Lotze, N. Bogdanoff, G. Reecht, L. Zhang, A. L. Briseno, and K. J. Franke, Physical Review B **100** (2019), 10.1103/physrevb.100.035410.

[8] R. C. d. C. Ferreira, A. Sagwal, J. Doležal, T. Neuman, and M. Švec, Nature Communications **16**, 6039 (2025).

[9] A. Rosławska, T. Neuman, B. Doppagne, A. G. Borisov, M. Romeo, F. Scheurer, J. Aizpurua, and G. Schull, Physical Review X **12**, 011012 (2022).
